# Comparative RNA-Seq Analysis Reveals Potentially Resistance-Related Genes in Response to Bacterial Canker of Tomato

**DOI:** 10.3390/genes12111745

**Published:** 2021-10-29

**Authors:** Leonardo I. Pereyra-Bistraín, Cesaré Ovando-Vázquez, Alejandra Rougon-Cardoso, Ángel G. Alpuche-Solís

**Affiliations:** 1 División de Biología Molecular, Instituto Potosino de Investigación Científica y Tecnológica A.C., San Luis Potosí 78216, Mexico; leonardo.pereyra@ipicyt.edu.mx; 2 Centro Nacional de Supercómputo, Instituto Potosino de Investigación Científica y Tecnológica A.C., Consejo Nacional de Ciencia y Tecnología, San Luis Potosí 78216, Mexico; cesare.ovando@ipicyt.edu.mx; 3 Laboratory of Agrigenomic Sciences, Universidad Nacional Autónoma de México, ENES-León, León 37689, Mexico

**Keywords:** arcanum, bacterial canker, *Clavibacter*, gene expression, RNA-Seq, tomato, transcriptome

## Abstract

Tomato is one of the most important crops for human consumption. Its production is affected by the actinomycete *Clavibacter michiganensis* subsp. *michiganensis* (*Cmm*), one of the most devastating bacterial pathogens of this crop. Several wild tomato species represent a source of natural resistance to *Cmm*. Here, we contrasted the transcriptomes of the resistant wild tomato species *Solanum arcanum* LA2157 and the susceptible species *Solanum lycopersicum* cv. Ailsa Craig, during the first 24 h of challenge with *Cmm*. We used three analyses approaches which demonstrated to be complementary: mapping to *S. lycopersicum* reference genome SL3.0; semi de novo transcriptome assembly; and de novo transcriptome assembly. In a global context, transcriptional changes seem to be similar between both species, although there are some specific genes only upregulated in *S. arcanum* during *Cmm* interaction, suggesting that the resistance regulatory mechanism probably diverged during the domestication process. Although *S. lycopersicum* showed enriched functional groups related to defense, *S. arcanum* displayed a higher number of induced genes related to bacterial, oomycete, and fungal defense at the first few hours of interaction. This study revealed genes that may contribute to the resistance phenotype in the wild tomato species, such as those that encode for a polyphenol oxidase E, diacyl glycerol kinase, TOM1-like protein 6, and an ankyrin repeat-containing protein, among others. This work will contribute to a better understanding of the defense mechanism against *Cmm*, and the development of new control methods.

## 1. Introduction

Tomato is one of the most important crops for human consumption. Its production can be affected by several phytopathogens. The actinomycete *Clavibacter michiganensis* subsp. *michiganensis* (*Cmm*) is the causal agent of the bacterial canker of tomato. This disease was described more than 100 years ago in Michigan, United States [1], and now it is present in almost all tomato-producing areas [2]. *Cmm* is considered one of the most devastating pathogens of this crop [3]. If disease spreading is not properly controlled, the tomato production can be severely affected in fields and protected agriculture. In Mexico, about 200 hectares of greenhouse crops were lost in 2006, reaching a 40 million dollars loss [4].

*Cmm* can infect tomato plants through wounds and natural entries such as stomata, hydathodes, and trichomes [5,6]. The typical transmission sources are infected tomato seeds, infected young seedlings, and residual plants in the soil [7,8,9]. Symptoms such as the unilateral wilting of leaves, cankers on stems, and the development of bird-eye spots in infected fruits are characteristic of the tomato bacterial canker [10]. Moreover, this pathogenic bacterium can survive in soil and plant debris for more than two years [11,12], which represents an infection source for subsequent farming seasons.

To date, there are no effective containment approaches against *Cmm*, and most of them are cultural practices and chemical control methods. Generally, several copper salts and antimicrobial compounds are used for chemical control, however, they can induce pathogen resistance, phytotoxicity, and phreatic surface pollution [13,14].

Several wild tomato species have been described as tolerant to *Cmm* infection, including *Solanum arcanum*, *Solanum habrochaites*, and *Solanum pimpinellifolium.* In these species, several quantitative trait loci have been associated with the defense response against this pathogenic bacterium [15,16,17]. *S. arcanum* LA2157 has been characterized with the highest degree of resistance against *Cmm* [15,18]. These wild tomato species can be considered a natural source of resistance that could be used for the improvement of the commercial tomato species *S. lycopersicum* by incorporation of resistance-related genes.

Previous studies have used differential gene expression analysis to identify genes probably associated with disease symptoms development during the *S. lycopersicum-Cmm* interaction. In one study, where microarrays were used, genes related to host-derived ethylene production were detected [19]. In another work, cDNA-AFLP was used to investigate the interaction of *Cmm* with two resistant wild tomato species and the susceptible tomato species *S. lycopersicum*. As a result, several differentially expressed genes, putatively associated with the tomato resistance, were identified [20]. Nevertheless, both methods may not represent the complete set of transcribed genes, due to technical limitations. RNA-Seq is a next generation sequencing technology that has been widely used in the last ten years. It allows one to obtain almost complete gene expression profiles in a specific condition, overcoming the limitations of other transcriptomic analysis tools, such as microarrays. Furthermore, RNA-Seq downstream analyses permit the classification and quantification of the whole diversity of RNA sequences, either by using an annotated genomic reference, or by de novo assembling the obtained sequences. This de novo strategy also allows for the detection of new genes and alternative splicing events [21,22,23].

In this study, we aimed to identify genes potentially related to the defense response against *Cmm* during the first hours of interaction, using RNA-Seq. For that purpose, we contrasted the global transcriptional changes of the non-model resistant wild tomato species *S. arcanum* LA2157 and the susceptible species *S. lycopersicum* cv. Ailsa Craig during the first 24 h of interaction with *Cmm*. This was performed using three different approaches of data analyses: mapping the RNA-Seq fragments to *S. lycopersicum* reference genome SL3.0 (MR); performing a semi de novo transcriptome assembly (STA); and a de novo transcriptome assembly (DA). Here, we report a multi-bioinformatics approach to compare the transcriptomes of two tomato species during the *Cmm* infection. Data provided in this study contribute to the screening of defense response genes on *Solanum* species.

## 2. Results

### 2.1. Infection of Tomato Plants with Cmm 1569

**Figure 1:**
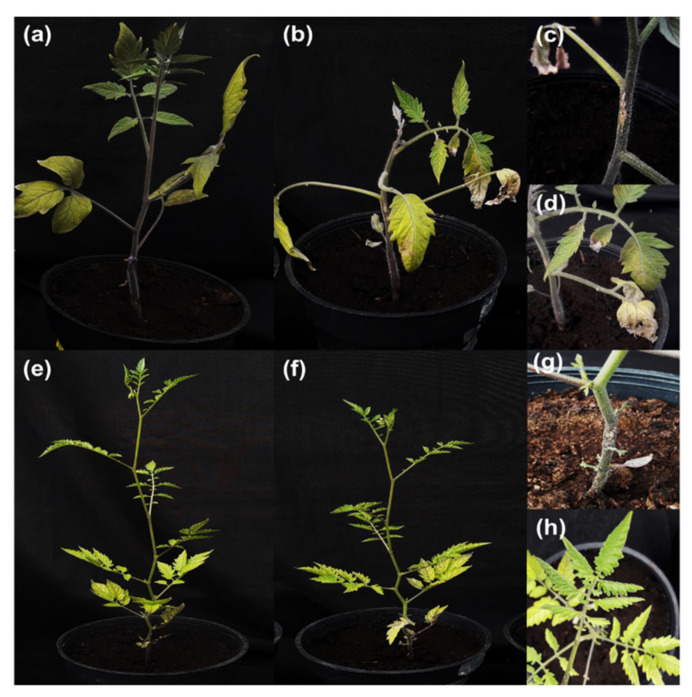
Development of disease symptoms in *S. lycopersicum* cv. Ailsa Craig and *S. arcanum* LA2157 infected with the *Cmm* 1569 strain. (**a**) *S. lycopersicum* plant 25 days after mock-inoculation. (**b**) *S. lycopersicum* 25 dpi, the plant showed the characteristic disease symptoms of bacterial canker. (**c**,**d**) *S. lycopersicum* 25 dpi, leaves showing unilateral wilt, and stem showing canker development. (**e**) *S. arcanum* plant 25 days after mock-inoculation. (**f**) *S. arcanum* 25 dpi. (**g**,**h**) *S. arcanum* 25 dpi, leaves and stem without disease symptoms.

To obtain the genetic material for the analysis of *S. lycopersicum* and *S. arcanum* LA2157 during the interaction with *Cmm* 1569, we collected leaves of infected plants at 0, 8, and 24 h post-inoculation (hpi). We followed the disease development symptoms in both tomato species. *S. lycopersicum* infected plants developed the characteristic disease symptoms at 25 days post-inoculation (dpi). The symptoms were the unilateral wilting of leaves, and the stem canker extending far from the inoculation site, whereas mock-inoculated *S. lycopersicum* control plants had normal stems and leaves (Figure 1a–d). In the resistant species *S. arcanum* LA2157, the infected plants did not show these typical disease symptoms. Instead, the inoculated plants only showed a small canker wound located in the inoculation site of each plant that did not spread through the stem, therefore, the phenotype of the *S. arcanum* LA2157 infected plants was very similar to the control plants (Figure 1e–h). In summary, both tomato species displayed the expected phenotype after *Cmm* inoculation.

### 2.2. Mappings Stats and Transcript Quantification

**Figure 2:**
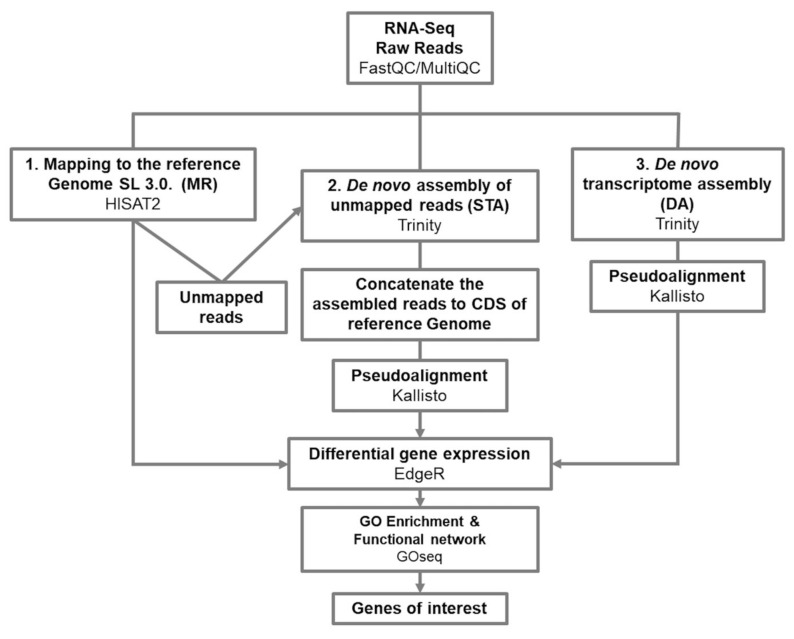
RNA-Seq data processing pipelines. The scheme summarizes the three strategies for data analysis: direct mapping to the reference genome of *Solanum lycopersicum* SL3.0, MR; (left), de novo assembly of unmapped reads, STA; (center), and de novo transcriptome assembly, DA (right). Processes and strategies are shown in bold and bioinformatic packages used for those processes are represented in light font.

We sequenced 18 libraries, resulting in fastq files ranging from 32–58 million reads of 150 bp each. We decided to analyze the sequencing data using three different strategies, which we will refer to as MR, STA, and DA (Figure 2; see Section 5). Simultaneously, these strategies allowed us to develop a comparative analysis among the three strategies, and obtain a consensus result suitable for detecting genes potentially related to a defense response against *Cmm*.

**Figure 3:**
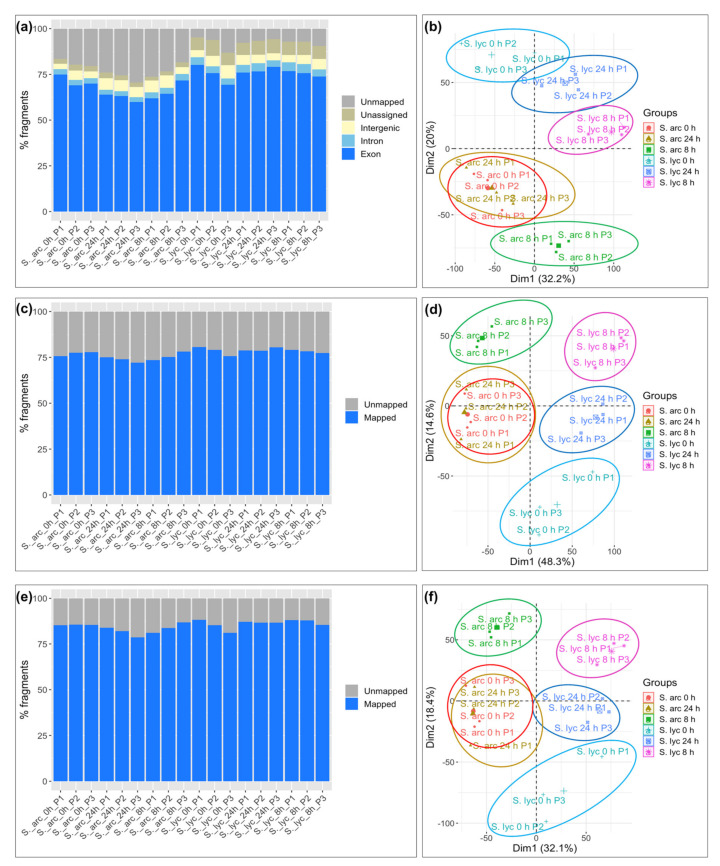
Mapping statistics of *S. arcanum* LA2157 and *S. lycopersicum* cDNA libraries, and sample similarity determined by principal component analysis (PCA). (**a**,**b**) Reads mapped to the *S. lycopersicum* SL3.0 reference genome (MR) and PCA. The average percentage of mapped reads was 77.3% for *S. arcanum*, and 92.4% for *S. lycopersicum*. (**c**,**d**) Mapping of sequence reads to the de novo-assembled unmapped reads, plus exonic regions of reference genome SL3.0 (STA) and PCA. The average percentage of mapped reads was 75.4% for *S. arcanum*, and 78.6% for *S. lycopersicum*. (**e**,**f**) Mapping of sequence reads to the de novo transcriptome assembly (DA) and PCA. The average percentage of mapped reads was 83.3% for *S. arcanum*, and 86.2% for *S. lycopersicum*. PCAs showed that biological replicates grouped correctly by study time (0, 8, and 24 hpi, accordingly) in all approaches. Count matrix adjusted by *Z*-score.

For the MR strategy, the percentage of mapped reads varied depending on the library, but on average, 77.3% of *S. arcanum* LA2157 and 92.4% of *S. lycopersicum* reads mapped to the reference genome (mapped to exonic, intronic, intergenic, and unassigned regions), resulting in a small percentage of unmapped reads to the reference (Figure 3a). In order to determine the sample similarity, we performed a principal component analysis (PCA). The biological replicates were grouped among them on each studied condition (i.e., 0, 8, and 24 hpi; Figure 3b). In the STA strategy, the average percentage of mapped reads for the *S. arcanum* species was 75.4%, and 78.6% for *S. lycopersicum* (Figure 3c). The mapping percentage of the *S. arcanum* libraries slightly decreased with this strategy, and noticeably diminished for the *S. lycopersicum* ones. This last decrease could have occurred because we only considered the coding regions from the reference genome and the de novo-assembled transcripts from unmapped reads for the STA strategy. The PCA obtained with this strategy presented the same distribution patterns for each group of biological replicates compared to the MR strategy (Figure 3d). With the DA pipeline, the average mapping percentage was 83.6% for *S. arcanum* LA2157 libraries, and 86.2% for *S. lycopersicum* libraries (Figure 3e). The grouping patterns in the PCA with the DA strategy agreed with the other two strategies (Figure 3f). Therefore, the application of either mapping approach did not affect the sample similarity of biological replicates. In parallel, we took advantage of the performed analyses to evaluate the computing time required for each strategy. For the de novo transcriptome, the assembly took ~279 h compared to ~25 h for the semi de novo transcriptome assembly and ~3 h for the mapping to the reference genome strategy (Table S1), pointing to considerable differences in the computing time required for each approach.

### 2.3. Differentially Expressed Genes in Response to Cmm 1569

We determined the number of differentially expressed genes (DEGs) in each strategy of analysis. The statistical significance was evaluated by establishing a false discovery rate (FDR) limit of 0.01 for data mapped to the reference genome SL3.0, and an FDR value of 0.1 for data mapped to the semi de novo and the de novo-assembled transcriptomes. The 0.1 FDR value was established considering that the reads mapped to 77,515 and 49,340 transcripts for the STA and DA approaches, respectively (Table S2), even though there are about 35,000 genes annotated in the tomato genome (one transcript per gene). Therefore, the reads could be assigned to several transcript isoforms coming from the same gene, and a restrictive FDR cutoff could result in the assignment of less DEGs. Table 1 provides the number of DEGs detected in each analysis. By setting up these FDR thresholds, we obtained comparable numbers of differential transcripts in all three strategies.

The entire dispersion patterns of DEGs are shown in Figure S1. The average percentage of up-regulated genes from 0 to 8 hpi in the three approaches was similar in *S. lycopersicum* and *S. arcanum* (7.46% compared to 6.57%, respectively), whereas the average percentage of down-regulated genes in *S. lycopersicum* was higher compared to *S. arcanum* species at this same time (9.28% compared to 6.08%, respectively). Regarding the 8 to 24 hpi transition time, the average percentage of induced and repressed genes was higher in *S. arcanum* compared to *S. lycopersicum* (Table 1).

**Figure 4:**
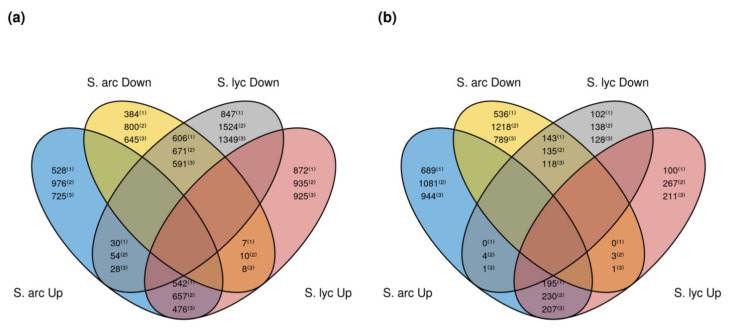
Venn diagrams showing DEGs contrasts between *S. lycopersicum* cv. Ailsa Craig and *S. arcanum* LA2157. (**a**) Contrasts of DEGs from 0 to 8 hpi transition. (**b**) Contrasts of DEGs from 8 to 24 hpi transition. (1) Mapping of reads to the reference genome SL3.0 (MR); FDR value 0.01. (2) Mapping to the semi de novo assembled transcriptome (STA); FDR value 0.1. (3) Mapping to the de novo assembled transcriptome (DA); FDR value 0.1.

Differences in induced and repressed genes in *S. arcanum* and *S. lycopersicum* could be seen as a starting point to search for genes involved in a defense response to *Cmm*. We detected shared genes between *S. lycopersicum* and *S. arcanum* LA2157 considering the DEGs and transition times (Figure 4). Regarding induced genes in *S. arcanum* and repressed in *S. lycopersicum*, 28 to 54 genes were detected from 0 to 8 hpi, indicating a similar number of genes in all strategies of analysis (Figure 4a). However, from the 8 to 24 hpi for shared induced genes in *S. arcanum* and repressed in *S. lycopersicum,* only one gene was detected by the DA strategy, and four genes by the STA one (Figure 4b). Depending on the strategy, 528 to 976 induced genes were detected exclusively in *S. arcanum* from 0 to 8 hpi (Figure 4a). At the same time, a similar number of induced genes was detected in *S. lycopersicum* only (around 900 genes in each analysis). Relative to the 8 to 24 hpi transition, between 689 and 1081 genes were induced in *S. arcanum* solely, and only from 100 to 267 in *S. lycopersicum* (Figure 4b). Regarding induced genes shared in both tomato species from 0 to 8 hpi, we detected 476 to 657 genes per strategy (Figure 4a). As for 8 to 24 hpi, around 200 genes were detected in each approach (Figure 4b). A similar number of repressed genes shared in both species, and time transitions, are visualized in Figure 4. Importantly, a higher number of repressed genes was detected solely in *S. lycopersicum* compared with *S. arcanum* from 0 to 8 hpi (Figure 4a), and this behavior was the opposite from 8 to 24 hpi (Figure 4b), which suggests a downregulation provoked by *Cmm* in *S. lycopersicum*. Remarkably, the overall results presented the same tendencies regardless of the used strategy.

### 2.4. Gene Ontology Term Enrichment Analysis in S. lycopersicum and S. arcanum LA2157

In order to determine the global transcriptional context of both tomato species in response to *Cmm*, we performed a gene ontology (GO) enrichment analysis considering the log fold change (LogFC) values and −log10 FDR values of DEGs. After extracting the enriched groups of each approach, we selected enriched GO terms that could be directly associated with an infection response caused by *Cmm*. In addition, enrichment scores of the selected functional groups of *S. lycopersicum* and *S. arcanum* were compared in order to detect which tomato species exhibited the higher enrichment. The complete enriched GO terms can be found in Figures S2–S4.

**Figure 5:**
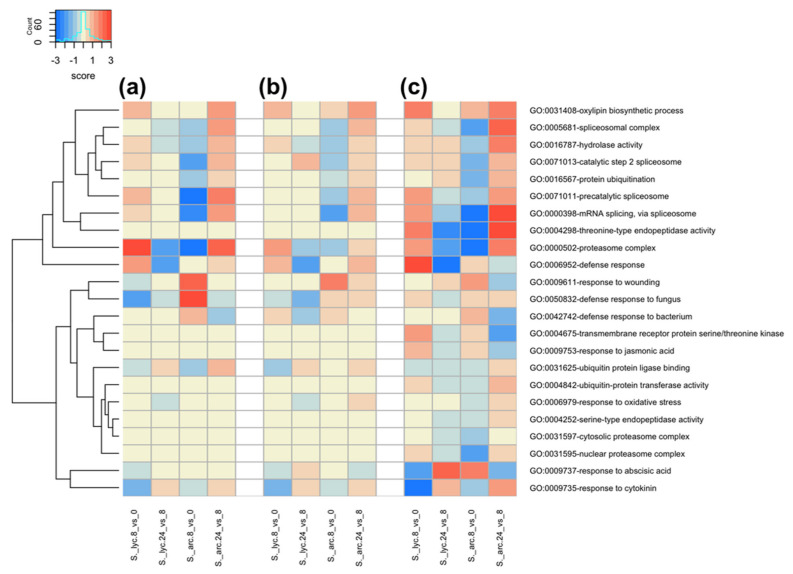
GO term enrichment analysis. Heatmaps representing the main enriched functional groups in each strategy of analysis, contrasting the enrichment level of each species. (**a**) Mapping to the reference genome SL3.0 (MR). (**b**) Mapping to the semi de novo-assembled transcriptome (STA). (**c**) Mapping to the novo-assembled transcriptome (DA). The score values were obtained using the LogFC and the −log10 of FDR of DEGs.

Several functional groups were enriched in both *S. lycopersicum* and *S. arcanum* in the three strategies of analysis, including hydrolase activity (GO:0016787), proteasome complex (GO:0000502), and response to cytokinin (GO:0009735). However, they were enriched differently either by induced or repressed genes, depending on the tomato species and time of study (Figure 5a–c). Similarly, we detected functional groups only enriched in one of the three strategies of analysis, such as nuclear proteasome complex (GO:0031595) and threonine type endopeptidase activity (GO:0004298), which were enriched only when we mapped the reads to the de novo-assembled transcriptome (Figure 5c). Thus, we observed that some functional groups were not enriched using the MR and STA strategies and could be only appreciated employing the DA approach.

Importantly, the more general functional group defense response (GO:0006952) was enriched in the three strategies of analysis. This functional group exhibited a higher enrichment by induced genes in *S. lycopersicum* from 0 to 8 hpi in all cases (Figure 5). However, the more specific functional groups corresponding with defense response to bacteria (GO:0042742), as well as with the defense response to fungus (GO:0050832), showed a stronger enrichment by induced genes in *S. arcanum* from 0 to 8 hpi. This behavior was consistent in all three analysis approaches (Figure 5).

### 2.5. DEGs Presumably Associated with the Defense Response against Cmm 1569

Using the results of all applied strategies, we aimed to obtain a list of genes potentially related to defense against *Cmm*. To do this, we first detected relevant genes in each approach considering the LogFC values, the comparisons (shared and unshared genes between the two tomato species), the GO term enrichment analysis, and the gene functional annotation. Then, we compiled the genes in a single list (Table 2). Genes that appeared in more than one approach (same gene identifier) were arbitrarily reported in one method only, to avoid redundancies. The complete gene expression lists can be observed in Tables S3–S8.

We identified DEGs induced only in *S. arcanum* from 0 to 8 hpi, such as genes that codify a polyphenol oxidase E (Solyc08g074620.3), an E3 ubiquitin protein-ligase (Solyc12g049330.2), a putative late blight resistance protein (Solyc05g012900.3), and a wall-associated receptor kinase-like (TRINITY_DN16553_c1_g3_i1). The last one also appeared from 8 to 24 hpi but repressed (Table 2). Likewise, induced genes in *S. arcanum* and repressed in *S. lycopersicum* were detected from 0 to 8 hpi, such as a gene that codifies a rust resistance kinase Lr10 (Solyc02g081500.3, which is repressed in *S. arcanum* from 8 to 24 hpi), a leucine-rich repeat receptor like serine/threonine protein kinase (Solyc11g017270.2), an unannotated gene with function related to serine type endopeptidase inhibitory activity (TRINITY_DN15795_c0_g2_i2), an ankyrin repeat-containing protein (Solyc08g062360.3, later repressed in *S. arcanum* from 8 to 24 hpi), an L-type lectin-domain containing receptor kinase (TRINITY_DN22346_c2_g3_i1, later induced in *S. lycopersicum* from 8 to 24 hpi), a mitogen-activated protein kinase 3 (TRINITY_DN15770_c0_g1_i7), and others (Table 2). Also, induced genes in both tomato species were detected from 0 to 8 hpi. However, some of them were strongly expressed in *S. arcanum* (Table 2), as is the case of a gene that codifies a probable receptor-like serine/threonine protein kinase (Solyc01g109590.3, which is repressed in *S. arcanum* from 8 to 24 hpi), and a class V chitinase (Solyc07g005090.3). We have also identified the top ten induced genes in *S. arcanum* with no transcripts assigned to *S. lycopersicum* detected with the DA approach (Table 3). Some of these induced genes may have an important role during the first hours of the defense response to *Cmm*, such as the genes that encode for a bidirectional sugar transporter SWEET2a (TRINITY_DN13677_c0_g1_i8) or the aquaporin PIP2-1 (TRINITY_DN15481_c0_g1_i3). Our study hosts many other genes of interest that can be identified by comparison of the two tomato species and the information provided in Tables S3–S8.

Several QTLs have been previously related to the resistance against *Cmm* [15,16,17]. We traced the regions spanning the QTLs in the *S. lycopersicum* genome, and we found 70 transcripts on chromosome 5, 42 on chromosome 7, and 2479 on chromosome 9 (Table S10). Interestingly, some of these transcripts were highly expressed in *S. arcanum* only at the first few hours of infection (Table 4). However, that is not the case in *S. lycopersicum*, where transcripts seemed to have little induction, or even downregulation. Moreover, the annotation of these transcripts correlates with previously published proteins involved in disease immunity.

### 2.6. RNA-Seq Validation Using DEGs Potentially Related to Resistance

In order to validate the expression profiles of differentially expressed genes identified by the three bioinformatics analyses, we selected five genes potentially related to resistance against *Cmm*. We carried out a qRT-PCR analysis of samples obtained from independent infection assays in *S. lycopersicum* and *S. arcanum* plants (different infected plants from those used in the RNA-Seq experiment). We considered only the infection times of 0 hpi and 8 hpi, since our results suggested that the most notable expression changes occurred in this interval of time after infection.

**Figure 6:**
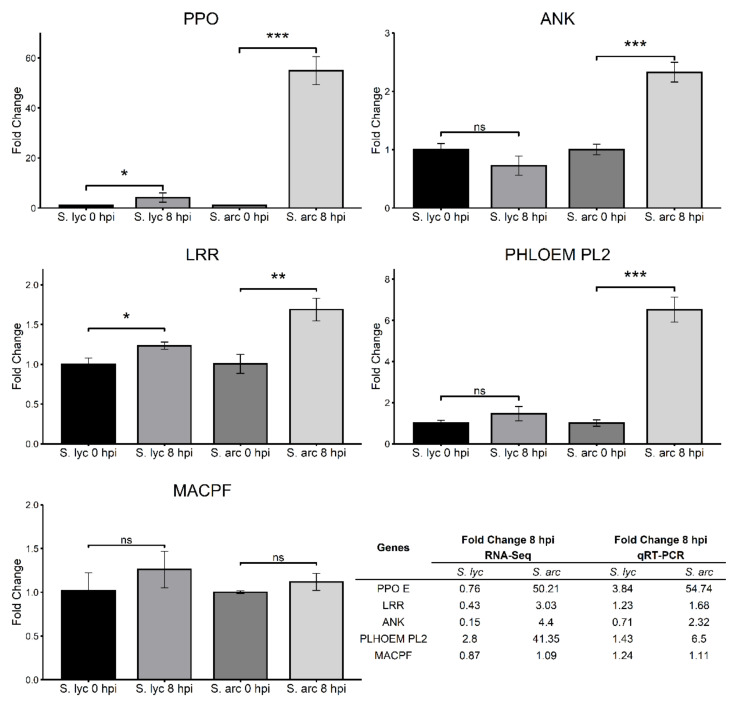
qRT-PCR analysis of selected DEGs. Genes analyzed: polyphenol oxidase E (PPO E; Solyc08g074620.3), leucine-rich repeat receptor-like serine-threonine-protein kinase (LRR; Solyc11g017270.2), ankyrin repeat-containing protein (ANK; Solyc08g062360.3.1), protein PHLOEM PROTEIN 2-LIKE A10 (PHLOEM PL2; Solyc02g092140.1), and MACPF domain-containing protein CAD1 (MACPF; Solyc10g085710.2). The expression levels were normalized to the actin expression (*ACTIN1*). Data represent fold change of gene expression 0 versus 8 hpi. The table compares the fold change values of RNA-Seq analysis versus fold change values from the qRT-PCR validation. The statistically significant differential gene expression between 0 hpi and 8 hpi was assayed by Student’s *t* test; unpaired. ns: >0.05. *: *p* ≤ 0.05. **: *p* ≤ 0.01. ***: *p* ≤ 0.001.

In our RNA-Seq analysis, four genes were detected as induced in *S. arcanum*. These genes were polyphenol oxidase E (PPO E; Solyc08g074620.3), leucine-rich repeat receptor-like serine-threonine-protein kinase (LRR; Solyc11g017270.2), the orthologous gene ankyrin repeat-containing protein (ANK; Solyc08g062360.3.1), and a protein PHLOEM PROTEIN 2-LIKE A10 (PHLOEM PL2; Solyc02g092140.1), and the expression patterns were alike with the qRT-PCR validation (Figure 6). The fifth gene selected is involved in plant immunity: an MACPF domain-containing protein CAD1 (MACPF; Solyc10g085710.2). According to our RNA-Seq results, this gene was not differentially expressed in *S. lycopersicum* nor in *S. arcanum*, and the expression levels were similar to those shown in the qRT-PCR analisys (Figure 6). Overall, the qRT-PCR results agreed with the comparative transcriptome analysis. The primers employed for qRT-PCR validation are shown in Table S9.

## 3. Discussion

We gained further insights about potentially resistance-related genes against *Cmm* as a result of the comparison of transcriptional changes in the resistant wild tomato species *S. arcanum* LA2157 and the susceptible species *S. lycopersicum* cv. Ailsa Craig. Additionally, mapping of the RNA-Seq raw data of *S. arcanum* species to the *S. lycopersicum* reference genome is a reliable strategy, despite the wild tomato species lacking a reference. Therefore, it is possible to use this MR approach for RNA-Seq analyses of wild-type tomato species. Nevertheless, the combination of our three bioinformatics pipelines (MR, STA, and DA) allowed us to obtain additional and complementary gene expression results.

### 3.1. From Mapping to a Reference Genome, to a De Novo Assembly Transcriptome; a Complementary Approach

Transcriptomic studies in non-widely-studied plants are a challenge as there is not enough data available. That is the case of the *S. arcanum* wild tomato species, where, at the time this research was conducted, there was not a good enough genome assembly sequence available for mapping. Usually, in an RNA-Seq analysis where there is no reference genome, the first alternative is to perform a de novo transcriptome assembly employing the preferred transcriptome assembling tool, such as Trinity, Oases, or SOAPdenovo-Trans [24,25,26]. However, the reference genome sequence of *S. lycopersicum,* which is relatively close to *S. arcanum* [27], has been available since 2012 [28]. Considering this phylogenetic relatedness, we decided to use the *S. lycopersicum* SL.3.0 reference genome for mapping the obtained reads of both tomato species (MR approach). Since this approach could deliver incomplete results, we decided to complement it and perform two other approaches, referred to as STA and DA (Figure 2).

A feature to highlight in an RNA-Seq analysis is the required computing time. As it has been reported, depending on the number of reads, a de novo transcriptome assembly with Trinity requires long running time periods and a high computing capacity [26], which could be disadvantageous when the latter is limited. According to our analysis, this approach consumed ~279 h compared with the MR approach, which only consumed ~2 h of computing time (Table S1). As our data demonstrate, the analysis based on the semi de novo assembly approach is a good alternative for bioinformatics transcriptome analysis, as it required an intermediate computing time of ~25 h (Table S1). Additionally, it was able to produce a higher number of transcripts in comparison to the other two strategies (Table S2).

Regarding the MR approach, average mapping percentages of *S. arcanum* LA2157 libraries fluctuated from around 70 to 80%, about 15% less than the average mapping percentage obtained in *S. lycopersicum*, which is a small difference given that they are different species. Furthermore, the amount of reads of both tomato species that correctly mapped to the reference genome varied from 70 to 90%, indicating good mapping proportions, which is in accordance with Sangiovanni et al. [29]. Therefore, considering the quality of the reference genome SL3.0, our results indicate a proper mapping for *S. arcanum* LA2157. Compared to MR, the STA approach improved the average mapping in the wild tomato species (Figure 3c). A similar pattern is present when reads were mapped to the de novo-assembled transcriptome (DA), with a higher mapping percentage of *S. arcanum* with respect to the STA (Figure 3e). Because of the good performance obtained with the Trinity assembler [30,31], we were able to discover new specific features that are not shared between these two related tomato species, or that were not available in the reference genome. These features can be found in the non-annotated genes that were only assigned to GO terms functions (Tables S3–S8), which could be analyzed in future research. Overall, we suggest that some missing information that cannot be obtained by the MR strategy could be compensated for by complementing the results with the DA and STA approaches.

The PCA led us to determine that samples cluster by species and by study condition in all approaches (Figure 3). Additionally, the PCA indicates a cyclic pattern of the biological replicates, since it is possible to observe an overlap between the 0 hpi samples and 24 hpi samples of *S. arcanum*, not observed in *S. lycopersicum* (Figure 3). The above results suggest a faster global response of *S. arcanum* to the *Cmm* infection in comparison to *S. lycopersicum*, which could have a delayed response, as has been previously proposed by Lara-Ávila et al. [20].

The total number of DEGs were similar regardless of the used approach. The STA strategy showed more DEGs than the DA strategy (Table 1), and this can be explained because the STA transcriptome is larger (harbors information of annotated genes from the reference genome SL3.0 and additional transcripts obtained after the assembly of unmapped reads; Table S2). Interestingly, in all approaches, we detected an approximately equal proportion of induced genes in both tomato species from 0 to 8 hpi (Table 1). Also, the proportion of repressed genes in *S. lycopersicum* compared to *S. arcanum* from 0 to 8 hpi was higher in all the three approaches (Table 1), which can be associated with the cyclic behavior observed in the PCA, and the suggested delayed response (Figure 3). Moreover, after contrasting DEGs of *S. lycopersicum* and *S. arcanum*, gene groups are comparable in the three approaches—these groups are shared between species, whereas other genes are induced or repressed in only one species (Figure 4). This information is useful to select those that are upregulated in the *S. arcanum* species only, and perhaps play an important role in bacterial plant defense. Overall, these analyses indicate that the number of DEGs detected in each approach is in concordance, and variations between one another are directly related to the number of genes/transcripts in the reference genome or assembled transcriptomes.

### 3.2. Global Transcriptional Profiling and Evidence of Resistance-Related Genes

According to GO term enrichment analysis, numerous functional groups revealed comparable patterns among MR, STA, and DA approaches in *S. arcanum* and *S. lycopersicum* (Figures S2–S4), suggesting that both tomato species share similarities in a global transcriptional context. Some of these enriched functions are directly related to plant defense (Figure 5). For example, compared with the resistant species *S. arcanum*, the general defense response functional group exhibited a higher enrichment level in the susceptible species *S. lycopersicum* from 0 to 8 hpi. This result agrees with previous evidence which demonstrated that even when *S. lycopersicum* is generating a defense response after sensing *Cmm*, it is not effective for counteracting the pathogen infection [19,32].

Importantly, during the transition from 8 to 24 hpi, the defense response group enrichment turned from induced genes to repressed genes in *S. lycopersicum*, whereas in *S. arcanum*, this functional group was enriched mainly by induced genes at 24 hpi (Figure 5). These findings are in accordance with a recent comparative transcriptome analysis between two different *Solanum* species and *Cmm* interaction, where the defense response functional group was not found in the susceptible tomato species *S. lycopersicum* var. filinta, although this experiment was done at four and eight days after *Cmm* inoculation (dai). In contrast, this functional group was enriched at 4 dai in the resistant tomato line *S. peruvianum* LA2157 [33]. Moreover, the functional groups of defense response specific to bacteria, as well as the defense response to fungi, were mainly enriched by induced genes from 0 to 8 hpi in *S. arcanum* (Figure 5), suggesting that *S. arcanum* is triggering a stronger defense response in comparison to *S. lycopersicum* after the challenge.

In addition, Basim et al. [33] reported a gene codifying for a TMV resistance protein N induced in *S. peruvianum* LA2157 at 4 dai. Interestingly, it seems that this gene is also participating during the first hours of the defense response to *Cmm*, as we also found transcripts from TMV resistance protein N genes mainly induced in *S. arcanum* from 0 to 8 hpi (Tables S3–S8). However, in our study, we did not find the *Sn-2* gene that has been associated with a defensive role against *Cmm* [34], probably because these genes may have a relevant defensive role several days after the interaction of resistant wild tomato species with *Cmm*, as has been recently showed in *S. peruvianum* LA2157 [33].

Although the results obtained by all strategies generally agreed, several functional groups showed enrichment in *S. arcanum* only in the STA and DA approaches. For example, in the MR approach, the oxylipin biosynthetic process was enriched only in *S. lycopersicum* from 0 to 8 hpi (Figure 5). However, in the same transition time, this functional group was enriched in *S. arcanum* together with *S. lycopersicum* when we applied the STA and DA approaches. In contrast, the response to wounding GO term was enriched from 0 to 8 hpi in *S. arcanum* in all approaches, but it also appeared enriched in *S. lycopersicum* from 8 to 24 h in the DA approach only (Figure 5). The enrichment of this group can be associated with the mechanical damage caused during *Cmm* inoculation.

According to our GO terms analysis, several functional groups that have been directly or indirectly associated with defense responses were enriched by induced and repressed genes. The oxylipin biosynthetic process GO term is defined as the chemical reactions and pathways resulting in the formation of oxylipins. Based on previous works that have shown the involvement of oxylipins in plant–pathogen interactions and wounding [35,36], the high enrichment of this group in our analysis may be due to the infection with *Cmm,* as well as the wounding caused during inoculation. In *Arabidopsis thaliana* leaves, oxylipins tend to accumulate to high levels during hypersensitive response to *Pseudomonas syringae* pv. tomato (*Pst*) expressing the avirulence gene *avRpm*1 [37]. Correspondingly, silencing of an α-dioxygenase gene, which is involved in oxylipin biosynthesis, enhances the susceptibility to several bacterial pathogens, and suppresses the hypersensitive response in *Capsicum annum* [38]. The cytokinin GO term was similarly enriched in both tomato species, particularly with induced genes from 8 to 24 hpi (Figure 5). Cytokinins (Ck) are plant growth hormones that affect plant immunity to several pathogens, such as *Rhodococcus fascians*. This pathogen produces Ck recognized by *A. thaliana* AHK3 and AHK4 receptors, leading to the development of disease symptoms [39]. Nonetheless, it has also been described that endogenous Ck promotes resistance in *A. thaliana* in response to *Pst*, which does not secrete Ck into the host [40]. It is likely that *Cmm* does not secrete Ck into tomato plants, suggesting that endogenous Ck are having a protective effect in the wild tomato. Other GO terms are enriched mainly in *S. arcanum*, such as mRNA splicing, protein ubiquitination, transmembrane receptor serine/threonine kinase, response to oxidative stress, ubiquitin ligase binding, etc. (Figure 5). These GO terms harbor traits that play several roles in the plant defense response [41,42,43,44]. Altogether, these results show some of the genes that may be involved in the defense response background of wild tomato species against *Cmm*.

Several genes are mainly induced in *S. arcanum* from 0 to 8 hpi in the MR approach, such as a gene that codifies to a PPO E. Polyphenol oxidases are involved in the oxidation of phenols to quinones. Previous studies have demonstrated a defensive role in transgenic tomato plants overexpressing a PPO of potatoes (*Solanum tuberosum*). Such transgenic plants exhibited an increased resistance to the pathogen *Pst*, reducing the bacterial growth, as well as severity of disease symptoms [45]. Moreover, downregulation of the PPO family in tomato, through the introduction of a chimeric antisense potato PPO cDNA, induced an increased susceptibility to *Pst*, suggesting that PPOs have a significant role in limiting disease development [46]. In the STA approach, we identified a non-annotated gene putatively related with serine-type endopeptidase inhibitory activity induced in *S. arcanum* from 0 to 8 hpi. Proteinase inhibitors are characterized as enzymes involved in several biological processes, including plant defense. Proteinase inhibitor StPI from a variety of *S. tuberosum* resistant to *Ralstonia solanacearum* is strongly induced during the first 6 to 12 h after the exposure with the pathogen and jasmonic acid treatment [47]. Likewise, transgenic tobacco lines over-expressing the multi-domain proteinase inhibitor NA-PI from *Nicotiana alata* and a β-hordothionin from barley are resistant to the fungal pathogen *Botritys cinerea* and the bacterial pathogen *Pseudomonas solanacearum* [48]. Therefore, the fact that we observed induced genes, particularly in *S. arcanum*, associated with PPOs and putative proteinase inhibitors during the challenge with *Cmm*, might indicate their relevance in this plant–pathogen interaction.

Our analysis showed DEGs encoding leucine-rich repeat receptor-like kinases (LRR-RLKs) in *S. arcanum* and *S. lycopersicum* species from 0 to 8 hpi (Table 2 and Tables S2–S8). This was an expected result, since several LRR-RLKs having a role in the defense response have previously been identified. For example, the NILR1 receptor is involved in resistance to parasitic nematodes [49]. In rice (*Oryza sativa*), the *Xa26* gene confers resistance against the bacterial pathogen *Xanthomonas oryzae* pv. *oryzae* (*Xoo*) [50]. Similarly, the overexpression of the *ERECTA* gene in *A. thaliana* var Ler displays an increased disease resistance to *R. solanacearum*, as indicated by reduced wilt symptoms and impaired bacterial growth [51]. In our study, the observed induction of this group of genes in both tomato species might indicate a role in the defense response against *Cmm*, and some specific members, such as LRR receptor-like serine/threonine-protein kinase gene products (e.g., Solyc11g017270.2 and TRINITY_DN14123_c3_g1_i2), could be particularly involved.

This study showed the induction of genes encoding proteins related to ankyrin repeat-containing domain, mostly in *S. arcanum* from 0 to 8 hpi. In plants, the ankyrin proteins belong to a large family of proteins involved in diverse functions, including plant defense. In *A. thaliana*, the ankyrin-repeat transmembrane protein BDA1 plays an important role in defense against bacteria. Loss of function mutations in the *BDA1* gene result in enhanced disease susceptibility to pathogen *Pst* DC3000, as well as in the nonpathogenic strain *Pst* DC30 *hrcC*, whereas a gain-of-function allele of *bda1* constitutively activated cell death and defense response [52]. Similarly, the XA21 binding protein 25 (XB25), a plant-specific ankyrin-repeat protein of rice, interacts with XA21, a protein that confers resistance to a broad spectrum of *Xoo*. In addition, the accumulation of XB25 is induced by *Xoo* infection. The repression of *Xb25* diminishes the accumulation of XA21, resulting in a compromised disease resistance, indicating that XB25 is required for maintaining XA21-mediated disease resistance [53]. ANK (Solyc08g062360.3.1) was one of the transcripts differentially expressed from 0 to 8 hpi in *S. arcanum*, suggesting that this gene could also be participating during the first hours of the defense response to *Cmm*.

Another interesting gene detected was a PHLOEM PROTEIN 2-LIKE A10 (PHLOEM PL2; Solyc02g092140.1) gene. *Cmm* destroys xylem vessels and phloem tissues as the bacteria multiply [10]. Induction of phloem proteins has been associated with diverse external stresses, including pathogenic attack. In our experiment, PHLOEM PROTEIN 2-LIKE A10 was induced in *S. arcanum* from 0 to 8 hpi (Figure 6), suggesting a protective role against *Cmm* infection.

We found several genes that were induced only in *S. arcanum* (Table 3). For instance, an aquaporin PIP2-1 upregulated at 8–24 h, a class of small, membrane channel proteins that can facilitate selective flux of various small molecules involved in numerous essential processes across membranes. Recently, evidence has pointed to aquaporins having a role in plant defense against pathogens [54]. After pathogen infection, apoplastic H_2_O_2_ is rapidly translocated into the cytoplasm across the plasma membrane (PM). A recent work demonstrated that AtPIP1;4, which is one of the PIP family members in *Arabidopsis*, is involved in the transport of H_2_O_2_ across the PM [55]. We also found a glyceraldehyde-3-phosphate dehydrogenase (GAPDH), which has been reported to have a role in immune responses to *Xanthomonas axonopodis* pv. *manihotis* in cassava [56], and a northern blot analysis showed the accumulation of cytosolic GAPDH gene transcripts in the leaves and stems of inoculated potato plants with an elicitor from the late blight fungal agent *Phytophthora infestans* [57]. In our analysis, a *SWEET2* gene induced in *S. arcanum* from 0 to 8 hpi. SWEET proteins possess an important role during plant–pathogen interactions. For example, in the roots of *Arabidopsis*, the loss-of-function *SWEET2* mutants are more susceptible to infection by the oomycete *Pythium irregulare* in comparison to *Arabidopsis* plants expressing *SWEET2*, suggesting that SWEET2 activity contributes to resistance to *P. irregulare* possibly by reducing the availability of glucose in the rhizosphere [58]. Another interesting transcript is one annotated as LNK1. It has been suggested that biotic stress responses are regulated by the circadian clock in plants, and that bacterial infection disrupts clock gene expression to attenuate immune responses. Recent research has demonstrated that infection with *Pseudomonas syringae* in wild-type (WT) plants downregulated the expression of several core clock genes 1 h post-infection, including all members of the night light-inducible and clock-regulated (LNK) gene family, and this effect was attenuated in eds4 mutant, which is a highly susceptible mutant. Furthermore, lnk mutants were more susceptible than the WT to *P. syringae* infection [59]. Another protein found only in *S. arcanum* was a γ aminobutyrate (GABA) transaminase 2. It has been described that the recognition of bacterial and fungal pathogens has proven to trigger GABA accumulation in several plant species, including tomatoes [60]. The accumulated GABA has been suggested to be transported to the mitochondria, where it undergoes deamination via GABA transaminase (GABAT) to be converted into succinic semialdehyde (SSA) [61].

For many years, geneticists have attempted to clone the resistance genes present in the various QTLs identified for *C. michiganensis* resistance in *S. arcanum*. Although some progress has been made, the regions spanning the QTLs are large, and, to our knowledge, a fine mapping has not been published yet. We have analyzed the presence of transcripts in the regions spanning some QTLs. We found 70 transcripts on chromosome 5, 42 on chromosome 7, and 2479 on chromosome 9 (Table S10). Interestingly, the most highly expressed transcript at 0–8 h (TRINITY_DN21279_c1_g1_i2) that has homology to the region spanning the QTL in chromosome 5 (Table 4), has the same annotation as (UPF0481 protein At3g47200) the one recently found on a high-density pepper linkage map, which is a possible candidate of a QTL on chromosome 5 also, and it is related to *P. capsici* resistance [62]. Another interesting transcript is a putative hyoscyamine 6-dioxygenase. These proteins generate free radical species, which is an early event associated with the hypersensitive response (HR). A putative hyoscyamine 6-dioxygenase was also found to be induced at the early stages of infection in a potato cultivar resistant to *Phytophthora infestans* [63].

On the region of chromosome 7, we found homology to a transcript annotated as a diacylglycerol kinase (DGK) (Table 4). These are very important signaling enzymes that phosphorylate diacylglycerol (DAG) to produce phosphatidic acid (PA). There are two pathways by which PA can be produced: through phospholipase D (PLD); and the coupled phospholipase C (PLC)/DGK route [64]. The PLC/DGK pathway seems to be responsible for the rapid accumulation of PA during exposure to pathogen elicitors from bacteria, fungi, or oomycetes [65]. Moreover, a critical reductive coenzyme (NADPH) is produced by DGK enzymes. An oxidative burst driven by NADPH oxidases takes place in most plant–pathogen interactions, especially in incompatible interactions. DGK enzymatic activity seems to be increased after elicitor treatment in tomato, tobacco, rice, and *Arabidopsis* plants, as well as upon pathogen infection. It has been suggested that DGK is the principal producer of plant PA, and that it is involved in the basal transcriptome regulation, the stimulation of callose accumulation in the apoplast, and the tolerance to the pathogen *Pseudomonas syringae* [66]. Moreover, the overexpression of the rice DGK gene (OsBIDK1) in tobacco improved its tolerance to tobacco mosaic virus (TMV) and *Phytophthora parasitica* var. *nicotianae* infections [67]. On chromosome 7, we also found an RPP13-like protein 1. RPP13 is an *Arabidopsis* resistance gene that confers resistance to *Hyaloperonospora arabidopsidis* [68], and RPP13-like proteins have been found in many other plants associated with disease resistance. In the region of chromosome 9, we found a TOM1-like protein differentially regulated. It has been suggested that TOM1-like proteins have a role in the passage of endocytosed ubiquitinated plasma membrane cargo, and act as gatekeepers for the sorting of protein degradation to the vacuole [69]. Silencing of a homologue of TOM1-L2 (TOL) in *Nicotiana benthamiana* has reduced bacterial growth, thus, it has been proposed that TOL proteins negatively regulate the plant immune response [70].

On the other hand, Coaker et al. [71] analyzed the proteome of a susceptible tomato line and two isogenic tomato lines infected with *Cmm* and containing the QTLs Rcm 5.1 and Rcm 2 identified in *S. habrochaites* LA408, which are proteins related to *Cmm* resistance. This study revealed a role of oxidative stress in response to *Cmm*, since three distinct superoxide dismutase enzymes (SOD) were differentially regulated among the genotypes analyzed. Interestingly, we also found transcripts from superoxide dismutase genes mainly induced in *S. arcanum* from 8 to 24 hpi by the three analysis approaches (Tables S3–S8), which supports the previous finding made by Coaker et al. [71].

We validated our RNA-Seq results by qRT-PCR assays of five different genes related to the functions discussed above: a gene encoding a polyphenol oxidase E; an ankyrin repeat-containing protein; a LRR receptor-like serine/threonine-protein kinase; a protein PHLOEM PROTEIN 2-LIKE A10; and a MACPF domain-containing protein CAD1. The qRT-PCR results agree with the gene expression profiles observed in the RNA-Seq (Figure 6), supporting the reproducibility of our results. The moderate variation in gene expression levels is due to the qRT-PCR sensitivity, together with the inherent biological variability resulting from two independent infection assays in two different tomato species (one for RNA-Seq, and one for qRT-PCR). Therefore, these results provide not only statistical significance, but also biological significance to our analyses.

Most importantly, despite showing enriched GO terms related to defense, *S. lycopersicum* is unable to overcome *Cmm* infection. Our results suggest a delayed response in *S. lycopersicum* compared with the wild *S. arcanum*. Moreover, *S. arcanum* seems to have more induced genes at the first few hours after inoculation related to bacterial, oomycete, and fungal defense. Our mapping results also suggest high conservation levels between both species. However, there are some specific genes only upregulated in *S. arcanum* during *Cmm* interaction, suggesting that the resistance regulatory mechanism probably diverged during the domestication process. However, we cannot rule out the possibility that those genes specific to *S. arcanum,* which we could only find in the de novo assembly, have an important contribution in the defense against *Cmm*.

## 4. Conclusions

Our results demonstrated not only the feasibility of mapping to a closely related species, but also the relevance of a de novo or a semi de novo approach. They also showed consistent results that revealed many interesting genes that contribute to the resistance phenotype. Additionally, a qRT-PCR assay confirmed differential gene expression on some of the candidates. Our functional analysis has unfolded a diversity of processes associated with the differential phenotype. The huge amount of data generated by this study will surely contribute to a deeper understanding of defenses against *Cmm*. However, further analysis of this defense signaling network, to find genetic variations and, possibly, divergent regulation, would be essential to unleash the resistance processes taking place in both species. Differentially expressed genes between resistant and susceptible varieties, and their functional analysis contribute to the search for new options for generating resistant tomato varieties.

## 5. Materials and Methods

### 5.1. Pathogenic Cmm Strain and Plant Material

*S. lycopersicum* cv. Ailsa Craig tomato seeds were obtained from the University of Nottingham (Nottingham, UK). *S. arcanum* LA2157 seeds were acquired from the Tomato Genetics Resource Center (TGRC, Davis, CA, USA). *Cmm* 1569 strain was isolated from a farming area in Chiapas, Mexico, and donated by Dr. Pablo Lara-Ávila.

### 5.2. Growth Conditions and Plant Infection

All experiments were carried out in controlled conditions, as described below. Before germination, all tomato seeds were sterilized in a 10% Extran solution for 15 min. Subsequently, three washes with deionized water and one wash with 70% ethanol solution were performed, followed by a 10% bleach solution wash for 10 min, and rinsing three times in deionized water. Tomato seeds were germinated in a commercial mix substrate (Sunshine Mix #3, Sun Grow Horticulture, Vancouver, BC, CA) and a bioclimatic chamber at 25 °C with a 16/8 h light and dark period. Two weeks later, plants were transferred to greenhouse conditions with a controlled minimum and maximum temperature of 20–28 °C, and a humidity of 55–60%.

Forty days after germination, both tomato species were infected with the pathogenic strain *Cmm* 1569. The bacterial cells were propagated in an 802-medium culture (2 g L^−1^ yeast extract, 1 g L^−1^ polypeptone and 0.92 g L^−1^ magnesium sulfate). For inoculation, three biological replicates per tomato species (*S. lycopersicum* cv. Ailsa Craig and *S. arcanum* LA2157) were inoculated in the first proximal internode above ground using an insulin syringe with a *Cmm* solution at a concentration of 5 × 107 CFU (OD = 0.2). Plants were sampled (true leaves next to the inoculation site) at 0, 8, and 24 hpi. Samples were immediately frozen in liquid nitrogen, and stored at −80 °C. The plants were maintained in greenhouse conditions until the appearance of the characteristic disease symptoms.

### 5.3. Cdna Library Preparation and RNA-Seq

The 18 samples (nine samples from each plant species) were sent to the Beijing Genome Institute (BGI. Shenzhen, China). After the total RNA extraction and DNase I treatment, magnetic beads with oligo (dT) are used to isolate mRNA (for eukaryotes), or by removing rRNAs from the total RNA (for prokaryotes). Mixed with the fragmentation buffer, the mRNA is fragmented into short fragments. Then, cDNA is synthesized using the mRNA fragments as templates. Short fragments are purified and resolved with EB buffer for end reparation and single nucleotide A (adenine) addition. After that, the short fragments are connected with adapters. The suitable fragments are selected for the PCR amplification as templates. During the QC steps, Agilent 2100 Bioanaylzer, and ABI StepOnePlus Real-Time PCR System are used in quantification and qualification of the sample library. TruSeq paired-end libraries were produced, and sequenced with Illumina Hiseq10X (Illumina, Inc., San Diego, CA, USA). Multiplex sequencing was performed, and the average targeted length obtained per read was 150 bp.

### 5.4. Map to the S. lycopersicum Genome SL3.0

This pipeline was designed to quantify gene expression using the *S. lycopersicum* genome SL3.0 as reference, available at the time this research was conducted (we employed a cluster with 18 FAT nodes and 400 GB in RAM with 32 processors; Table S1). Raw reads quality assessment was performed using the program FastQC (https://www.bioinformatics.babraham.ac.uk/projects/fastqc/, accessed on 20 April 2021). A summary of all FastQC reports was performed with MultiQC [72]. The paired-end reads of each sample were mapped to *S. lycopersicum* genome version SL3.0 (https://solgenomics.net/organism/Solanum_lycopersicum/, accessed on 20 April 2021) with HISAT version 2.1.0 [73] using default parameters and the non-deterministic option. Mappings were sorted with Samtools version 1.3 [74]. Mapping results summaries were analyzed with an in-house R script (https://github.com/cesaremov/Solanum_Arcanum-Lycopersicum, accessed on 20 April 2021). Fragment quantification assignment to exons per gene identifier was performed with FeatureCounts version 1.6.2 [75]. Quantification results were analyzed and processed with an in-house R script to obtain counts and counts per million (cpm) tables. A principal component analysis was performed using cpm tables as input to *prcomp* function from the R stats package using the transcripts per million matrices obtained in each strategy as input (count matrix adjustment by Z-score function) [76]. A counts table was used as input to the differential expression analysis with the R package EdgeR [77]. The counts table was filtered to keep only transcripts with cpm ≥ 4 in at least nine samples. Dispersion was estimated with the EdgeR function *estimateDisp* with default parameters. Subsequently, the data was fitted to the negative binomial distribution with the EdgeR function *glmQLFit* using default parameters. We compared the data of *S. arcanum* from 0 to 8 hpi with the data of *S. lycopersicum* from the same time transition; and data of *S. arcanum* from 8 to 24 hpi with the data of *S. lycopersicum* from the same time transition. The EdgeR function *glmQLMFTest* was used to compare between conditions. Multiple testing correction was performed using the *p.adjust* function from R base.

To assess GO term enrichment [78], a logistic classification model defined by the generalized linear model (GLM) was employed. The *glm* function was used from the R stats package. The formula provided to the GLM was *index* ~ *statistics*, where the index is a Boolean index indicating if genes belong to a GO term. The statistics were defined by the *score* = *sign*(*logFC*)* − log10 (*p.value*) for each species. Weights in GLM were defined as the ratio of genes not in GO term to genes in GO term. The link function was set to *logit.* To assess GO term enrichment when both tomato species are competing, we used GLM as previously mentioned, but used both species scores in the same model. The formula for the model with species competing was: *index* ~ *statistic_S._arcanum + statistic_S._lycopersicum*. Each statistic is defined, as before, with scores of each species in the same model.

### 5.5. Map to the Semi De Novo Transcriptome

In this pipeline, the paired-end reads not mapped to the genome were assembled. All unmapped paired reads were gathered into one single sequence file. These unmapped reads were assembled using Trinity version 2.6.6 with default parameters to obtain unmapped transcripts [26]. The spliced junction mapped fragments were used to assemble mapped transcripts with StringTie [79] (see first pipeline). Unmapped and mapped assembled transcripts were combined to get a unique *semi* de novo transcriptomic reference. Coding regions were predicted using Transdecoder (https://github.com/TransDecoder/TransDecoder/wiki, accessed on 20 April 2021). This semi de novo assembly was functionally annotated using the *autoTrinotate.pl* program provided by Trinotate (https://github.com/Trinotate/Trinotate.github.io/wiki, accessed on 20 April 2021) with the programs BLAST and Hmmer [80]. The databases SwissProt (https://web.expasy.org/docs/swiss-prot_guideline.html, accessed on 20 April 2021) and Pfam [81] were used for the functional annotation. Each transcript could have one or more of the following functional annotations: blastx; blastp; Pfam; eggnog; or gene ontology. All RNA-Seq paired-end reads for each sample were mapped to this semi de novo transcriptomic reference using Kallisto version 0.42.4 [82]. The mapping results were analyzed as in pipeline one. We used Kallisto pseudo-counts (TPM) as input to differential expression analysis with the EdgeR R package. Subsequently, we used the same steps from the first pipeline to perform differential expression analysis.

### 5.6. Map to the De Novo Transcriptome

All forward and reverse paired-end fastq format files were gathered to get only a pair of forward and reverse fastq files using in-house programs. This pair was used as input for Trinity version 2.6.6 [24] with default parameters, and a minimum transcript length of 500 nucleotides. The result of Trinity was a de novo transcriptome assembly, which was used as reference in this third pipeline. Functional annotation was performed in the same way as in the second pipeline. The paired-end reads were mapped to this de novo transcriptome using Kallisto version 0.42.4 [82] with default parameters. As in the previous two pipelines, in-house R programs were applied to analyze mapping results. As mentioned in pipeline two, we got Kallisto quantifications for the de novo (third) pipeline with in-house R programs. The same steps from the first pipeline were followed to perform differential expression analysis.

### 5.7. Experimental Approach to Find Transcripts Showing Upregulation in S. arcanum Located at the Regions Spanning QTLs Associated with Resistance

The regions spanning the QTLs in chromosomes 5 (44,803,914–52,431,431), 7 (1,319,683–1,554,620), and 9 (1,753,139–2,313,950), published on Sen [83], were used as a database from the *S. lycopersicum* reference genome SL3.0 to do a blast search against the transcripts obtained from the de novo assembly of *S. arcanum* reads.

### 5.8. Gene Expression Validation with qRT-PCR Analysis

Total RNA from infected plants was used for the synthesis of single-stranded cDNA using SuperScript II Reverse Transcriptase and oligo dT (Invitrogen, Carlsbad, CA, USA). The cDNA samples were amplified with gene-specific primers designed in Primer 3.0 [84]. Possible dimers and heterodimers were predicted with Beacon designer tool (Premier Biosoft International, Palo Alto, CA, USA; http://www.premierbiosoft.com/qOligo/Oligo.jsp?PID=1, accessed on 20 April 2021). Possible amplicon secondary structures were detected with UNAFold tool (IDT Company, Coralville, IA, USA; https://www.idtdna.com/site/account/login?returnurl=%2FUNAFold, accessed on 20 April 2021). Quantification was performed on an Applied Biosystems, 7500 fast real-time-PCR system version 2.0 (Applied Biosystems, Foster City, CA, USA). The biological replicates of each condition were pooled together. The qRT-PCR reaction was carried out in 96-well plates, and the thermal cycling was: 94 °C, 5 min; 40 cycles of 94 °C, 25 s; 60 °C, 30 s. Experiments were carried out using Fast SYBR Green Master Mix (Applied Biosystems, Foster City, CA, USA). Expression levels were normalized to the *ACTIN1* housekeeping gene. Fold changes in RNA expression were determined using threshold cycles by the ΔΔCT method [85]. For each experiment, the qRT-PCR was performed in triplicates, and the presented values represent the average and standard deviation.

## Figures and Tables

**Table 1:** Differentially expressed genes identified in each strategy.

Plant Species.	0–8 hpi		8–24 hpi		Genes/Transcripts
Reference Genome SL3.0	Up	Down	Up	Down	Total
*S. lycopersicum*	1421	1483	295	245	14,477 FDR 0.01
*S. arcanum* LA2157	1100	997	884	679	
Semi de novo assembly					
*S. lycopersicum*	1602	2249	500	277	24,741 FDR: 0.1
*S. arcanum* LA2157	1687	1481	1315	1356	
De novo assembly					
*S. lycopersicum*	1409	1968	419	247	23,112 FDR: 0.1
*S. arcanum* LA2157	1229	1244	1152	908	
Average percentage of DEGs					
*S. lycopersicum*	7.46	9.28	1.95	1.29	
*S. arcanum* LA2157	6.57	6.08	5.46	4.69	
*p*-value	1.0	0.1	0.1	0.1	

Up: induced genes. Down: repressed genes. FDR: false discovery rate.

**Table 2:** Selected DEGs potentially related to resistant response against *Cmm*.

Gene/Transcript ID	Description	*S. arc* LogFC (0–8 hpi)	*S. lyc* LogFC (0–8 hpi)	*S. arc* LogFC (8–24 hpi)	*S. lyc* LogFC (8–24 hpi)
Reference genome					
Solyc06g074030.1	CCR4-associated factor 1 homolog 9	3.14 *	−3.85 *	−2.16	−1.07
Solyc06g071280.3	Protein EDS1B	2.32 *	−1.66 *	−1.73 *	−0.46
Solyc02g081500.3	Rust resistance kinase Lr10	2.62 *	−1.87 *	−1.80 *	−0.23
Solyc02g092140.1	Protein PHLOEM PROTEIN 2-LIKE A10	5.37 *	1.49 *	−2.96 *	−1.47
Solyc08g074620.3	Polyphenol oxidase E	5.65 *	−0.38	0.07	3.03
Solyc05g051530.3	ABC transporter G family member 11	5.48 *	−0.04	−4.90 *	−2.55
Solyc11g017270.2	Leucine-rich repeat receptor-like serine/threonine-protein kinase	1.60 *	−1.20 *	−0.53	0.71
Solyc08g080830.3	Probable LRR receptor-like serine/threonine-protein kinase	1.05 *	0.48	−0.69 *	−0.23
Solyc12g009140.2	Proteasome subunit α type-6-B	−0.83 *	−0.31	0.84 *	0.22
Solyc01g109590.3	Probable receptor-like serine/threonine protein kinase	2.65 *	1.58 *	−2.16 *	−1.09
Semi de novo assembly					
TRINITY_DN15795_c0_g2_i2	Serine-type endopeptidase inhibitor activity	6.19 *	−1.30 *	0.97	0.04
Solyc08g062360.3	Ankyrin repeat-containing protein	2.14 *	−2.67 *	−2.70 *	−0.88
Solyc06g066370.3	WRKY transcription factor WRKY24	1.33 *	−1.38 *	−0.96	0.51
Solyc12g049330.2	E3 ubiquitin-protein ligase SPL2	6.65 *	−0.05	−1.15	−0.06
Solyc05g012900.3	Putative late blight resistance protein homolog R1B-23	5.36 *	−1.62	−1.03	0.85
Solyc07g005090.3	Class V chitinase	2.84 *	1.65 *	0.83	0.07
Solyc06g083390.3	RPM1-interacting protein 4	1.29 *	−1.55 *	−0.74	−0.31
Solyc10g008010.3	Proteasome subunit α type-2	−0.67	0.10	1.02 *	−0.09
Solyc12g007110.2	Proline-rich receptor-like protein kinase PERK1	1.36 *	0.44	−0.81 *	0.49
De novo assembly					
TRINITY_DN22346_c2_g3_i1	L-type lectin-domain containing receptor kinase	2.44 *	−2.08 *	0.91	2.82 *
TRINITY_DN19315_c2_g1_i2	E3 ubiquitin-protein ligase RMA1H1	2.94 *	1.84	−0.75	0.89
TRINITY_DN17399_c2_g1_i1	NDR1/HIN1-like protein 2	4.54	−3.19 *	−0.57	−1.05
TRINITY_DN16733_c1_g1_i7	Annexin D4	2.72 *	−5.55 *	−1.32	1.74
TRINITY_DN15770_c0_g1_i7	Mitogen-activated protein kinase 3	2.12 *	−1.74 *	−0.54	−0.35
TRINITY_DN16314_c0_g3_i2	NAC domain-containing protein 2	2.30 *	−2.04 *	−0.56	1.16
TRINITY_DN22670_c0_g2_i2	Leucine-rich repeat receptor-like serine/threonine-protein kinase	1.67	−6.48 *	−0.51	5.47
TRINITY_DN16553_c1_g3_i1	Wall-associated receptor kinase-like 20	3.20 *	0.86	−1.85 *	−0.05
TRINITY_DN20729_c2_g1_i1	Protein EDS1	2.09 *	−1.58 *	−1.83 *	−0.44
TRINITY_DN14123_c3_g1_i2	Probable receptor-like protein kinase	1.75 *	1.26 *	−2.12 *	−0.72

LogFC values of genes differentially expressed are highlighted with an asterisk *.

**Table 3:** Top ten genes induced in *S. arcanum* with no transcripts assigned to *S. lycopersicum* after DA approach.

Gene/Transcript ID	Description	*S. arc* LogFC
0–8 hpi		
TRINITY_DN14010_c2_g1_i5	Tetraspanin-3	4.48
TRINITY_DN24943_c4_g1_i9	Linoleate 13S-lipoxygenase 2-1	3.88
TRINITY_DN18887_c0_g4_i2	UDP-glycosyltransferase 85A8	3.80
TRINITY_DN16833_c0_g1_i15	Unknown	3.44
TRINITY_DN13677_c0_g1_i8	Bidirectional sugar transporter SWEET2a	3.16
TRINITY_DN23205_c0_g6_i3	Ethylene-responsive transcription factor 1	2.95
TRINITY_DN23169_c1_g1_i1	γ aminobutyrate transaminase 2	2.78
TRINITY_DN19385_c0_g1_i7	Heavy metal-associated isoprenylated plant protein 32	2.63
TRINITY_DN18065_c0_g1_i1	Allene oxide synthase 2 chloroplastic	2.48
TRINITY_DN20602_c0_g1_i4	Ethylene-responsive transcription factor 5	2.45
8–24 hpi		
TRINITY_DN21371_c0_g1_i15	Stromal 70 kDa heat shock-related protein	6.39
TRINITY_DN16003_c0_g1_i7	Glyceraldehyde-3-phosphate dehydrogenase	3.67
TRINITY_DN15481_c0_g1_i3	Aquaporin PIP2-1	3.26
TRINITY_DN17153_c0_g1_i7	Heat shock cognate 70 kDa protein 2	3.24
TRINITY_DN23165_c1_g1_i17	RuBisCO large subunit-binding protein subunit α	3.15
TRINITY_DN24736_c3_g1_i8	Protein LNK1	2.74
TRINITY_DN17225_c0_g1_i11	Protein WVD2-like 7	2.7
TRINITY_DN23140_c2_g1_i7	Ferric reduction oxidase 6	2.65
TRINITY_DN25011_c3_g1_i11	Heat shock 70 kDa protein 15	2.52
TRINITY_DN25230_c3_g1_i22	Serine/arginine-rich splicing factor SC35	2.45

**Table 4:** Transcripts showing upregulation in *S. arcanum* located at the regions spanning QTLs associated with resistance.

Chromosome of QTL Spanning Region	Annotation	Transcript ID	*S._arc* LogFC		*S._lyc* LogFC	
			0–8 hpi	8–24 hpi	0–8 hpi	8–24 hpi
Ch5	UPF0481 protein At3g47200	TRINITY_DN21279_c1_g1_i2	6.90	−1.33	0.35	−1.33
Ch5	Hyoscyamine 6-dioxygenase	TRINITY_DN19945_c3_g1_i7	4.37	0.64	0.65	0.64
Ch7	Diacylglycerol kinase 7	TRINITY_DN20884_c0_g1_i10	5.82	0.26	0.45	−0.34
Ch7	Putative disease resistance RPP13-like protein 1	TRINITY_DN22407_c0_g2_i3	1.69	−1.12	0.21	−0.30
Ch9	TOM1-like protein 6	TRINITY_DN22806_c1_g1_i2	10.52	−0.07	−0.74	0.45

## Data Availability

Raw and processed data are available from the Gene Expression Omnibus (GEO), National Center for Biotechnology Information (NCBI) under the accession GSE152330.

## Supplementary Materials

The following materials are available online at https://www.mdpi.com/article/10.3390/genes12111745/s1, Figure S1. MA plots of DEGs per analysis approach. FDR 0.01 for MR analysis; FDR 0.1 for STA and DA analysis approaches. (**a**) Differentially expressed genes dispersion from 0 to 8 hpi. (**b**) Differentially expressed genes dispersion from 8 to 24 hpi. Red dots: repressed genes. Blue dots: induced genes. x-axis = mean average abundance (Log 2 CPM, counts per million); y-axis = log ratio (Log 2 FC). Figure S2. GO terms enrichment resulting from MR analysis. (**a**) Enriched functional groups in biological process ontology (BP). (**b**) Enriched functional groups in cellular component ontology (CC). (**c**) Enriched functional groups in molecular function ontology (MF). GO term enrichment was performed using a logistic regression comparing scores of the genes/transcripts. Scores were calculated from LogFC and the −log10 of FDR values. Figure S3. GO terms enrichment resulting from STA analysis. (**a**) Enriched functional groups in biological process ontology (BP). (**b**) Enriched functional groups in cellular component ontology (CC). (**c**) Enriched functional groups in molecular function ontology (MF). GO term enrichment was performed using a logistic regression comparing scores of the genes/transcripts. Scores were calculated from LogFC and the −log10 of FDR values. Figure S4. GO terms enrichment resulting from DA analysis. (**a**) Enriched functional groups in biological process ontology (BP). (**b**) Enriched functional groups in cellular component ontology (CC). (**c**) Enriched functional groups in molecular function ontology (MF). GO term enrichment was performed using a logistic regression comparing scores of the genes/transcripts. Scores were calculated from LogFC and the −log10 of FDR values. Table S1. Average computing time consumed per strategy. Table S2. Main features of generated transcriptomes. Table S3. MR approach 0–8 hpi. Table S4. MR approach 8–24 hpi. Table S5. STA approach 0–8 hpi. Table S6. STA approach 8–24 hpi. Table S7. DA approach 0–8 hpi. Table S8. DA approach 8–24 hpi. Table S9. List of primers employed for qRT-PCR validation. Table S10. Predicted transcripts spanning QTL regions on *S. lycopersicum* genome.
