# Supplementary material for: Comparative RNA-Seq Analysis Reveals Potentially Resistance-Related Genes in Response to Bacterial Canker of Tomato
Source: Genes (Basel). 2021 Oct 29;12(11):1745. doi: 10.3390/genes12111745 (PMC8618811; doi:10.3390/genes12111745)
Supplement: Supplementary file 1 [file genes-12-01745-s001.zip › Supplementary materials (Figures S1-S4_Tables S1_S2_S9).pdf]

Comparative RNA-Seq analysis reveals potentially resistance-related genes in response to bacterial canker of tomato

Authors. Leonardo I. Pereyra-Bistraín[1,+], Cesaré Ovando-Vázquez[2,+], Alejandra Rougon-Cardoso[3,*], Ángel G. Alpuche-Solís[1,*].

[1]División de Biología Molecular, Instituto Potosino de Investigación Científica y Tecnológica A.C., San Luis Potosí, México.

[2]Centro Nacional de Supercómputo, Instituto Potosino de Investigación Científica y Tecnológica A.C., Consejo Nacional de Ciencia y Tecnología, San Luis Potosí, México.

[3]Laboratory of Agrogenomic Sciences, Universidad Nacional Autónoma de México, ENES-León, León, México.

*Corresponding authors: alpuche@ipicyt.edu.mx; arougon@enes.unam.mx

[+]These authors contributed equally to this work

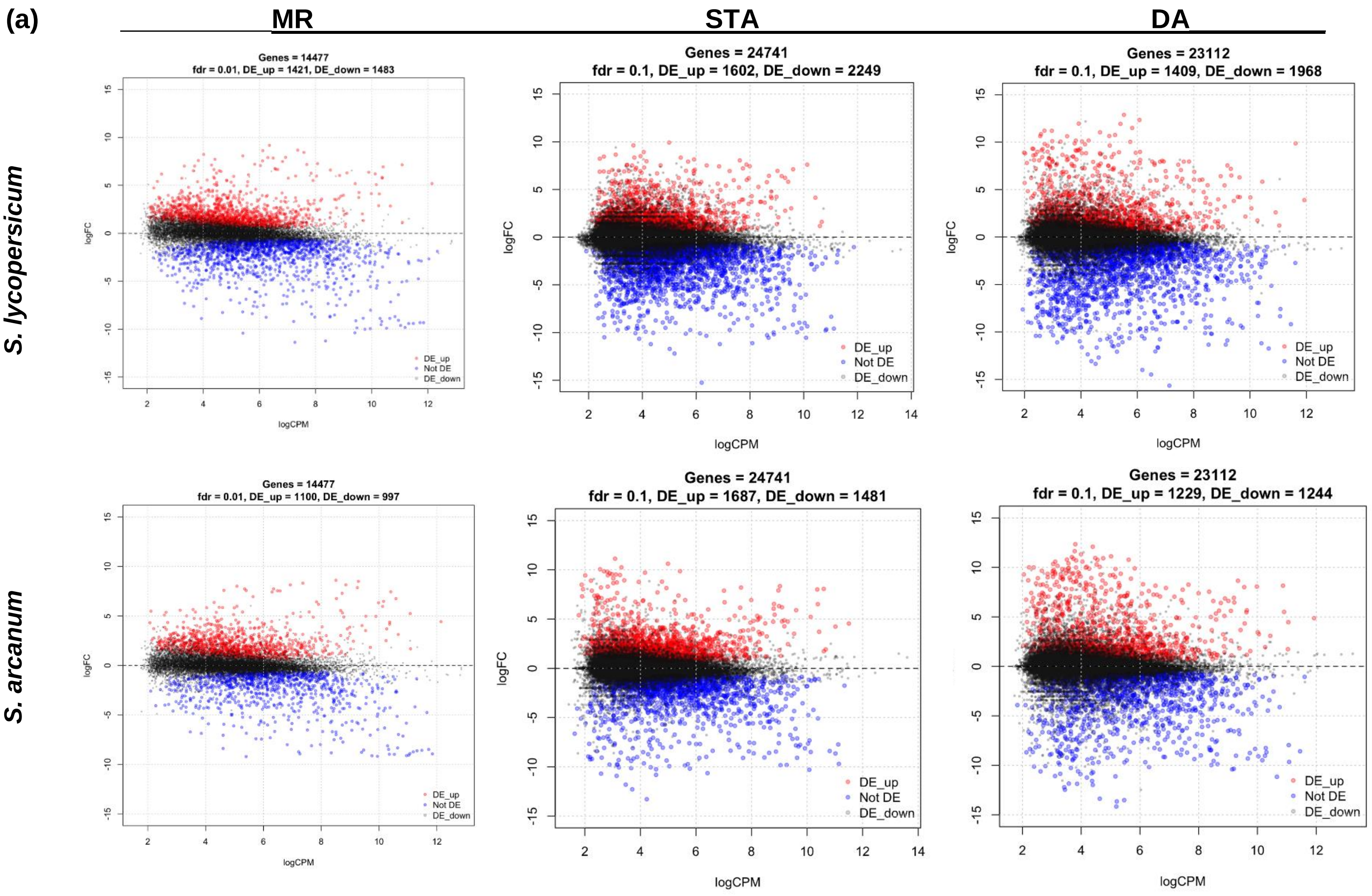

(a)
MR
STA
DA
S. lycopersicum
S. arcanum
Genes = 14477
fdr = 0.01, DE_up = 1421, DE_down = 1483
Genes = 24741
fdr = 0.1, DE_up = 1602, DE_down = 2249
Genes = 23112
fdr = 0.1, DE_up = 1409, DE_down = 1968
Genes = 14477
fdr = 0.01, DE_up = 1100, DE_down = 997
Genes = 24741
fdr = 0.1, DE_up = 1687, DE_down = 1481
Genes = 23112
fdr = 0.1, DE_up = 1229, DE_down = 1244
logFC
logCPM
DE_up
Not DE
DE_down

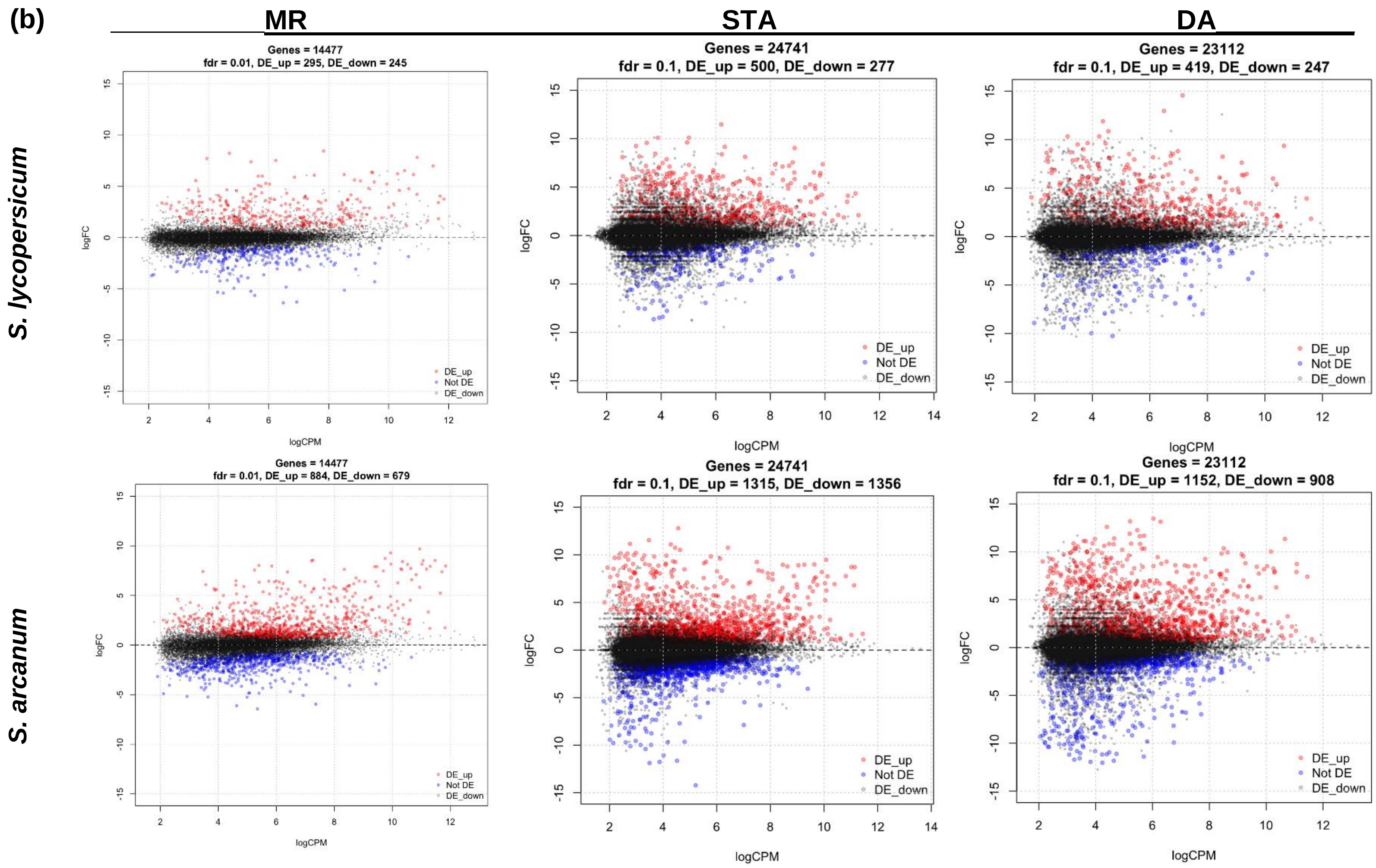

Figure S1. MA plots of DEGs per analysis approach. FDR 0.01 for MR analysis; FDR 0.1 for STA and DA analysis approaches. (a) Differentially expressed genes dispersion from 0 to 8 hpi. (b) Differentially expressed genes dispersion from 8 to 24 hpi. Red dots: repressed genes; blue dots: induced genes. x-axis = mean average abundance (Log 2 CPM, counts per million); y-axis = log ratio (Log 2 FC).

(a)

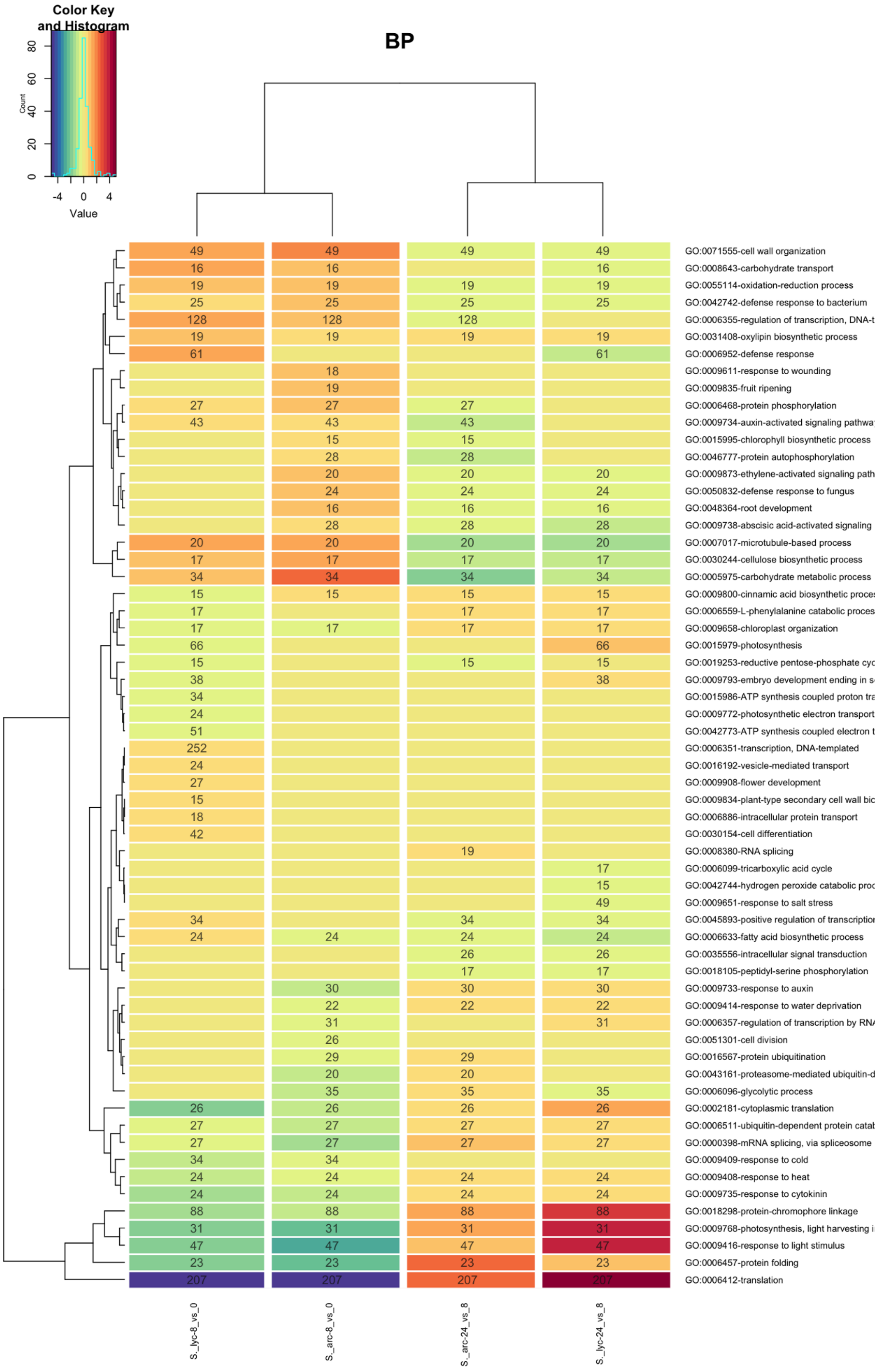
Color Key
and Histogram
Count
Value
BP
GO:0071555-cell wall organization
GO:0008643-carbohydrate transport
GO:0055114-oxidation-reduction process
GO:0042742-defense response to bacterium
GO:0006355-regulation of transcription, DNA-t
GO:0031408-oxylipin biosynthetic process
GO:0006952-defense response
GO:0009611-response to wounding
GO:0009835-fruit ripening
GO:0006468-protein phosphorylation
GO:0009734-auxin-activated signaling pathwa
GO:0015995-chlorophyll biosynthetic process
GO:0046777-protein autophosphorylation
GO:0009873-ethylene-activated signaling path
GO:0050832-defense response to fungus
GO:0048364-root development
GO:0009738-abscisic acid-activated signaling
GO:0007017-microtubule-based process
GO:0030244-cellulose biosynthetic process
GO:0005975-carbohydrate metabolic process
GO:0009800-cinnamic acid biosynthetic proce
GO:0006559-L-phenylalanine catabolic proces
GO:0009658-chloroplast organization
GO:0015979-photosynthesis
GO:0019253-reductive pentose-phosphate cyc
GO:0009793-embryo development ending in s
GO:0015986-ATP synthesis coupled proton tra
GO:0009772-photosynthetic electron transport
GO:0042773-ATP synthesis coupled electron t
GO:0006351-transcription, DNA-templated
GO:0016192-vesicle-mediated transport
GO:0009908-flower development
GO:0009834-plant-type secondary cell wall bic
GO:0006886-intracellular protein transport
GO:0030154-cell differentiation
GO:0008380-RNA splicing
GO:0006099-tricarboxylic acid cycle
GO:0042744-hydrogen peroxide catabolic proc
GO:0009651-response to salt stress
GO:0045893-positive regulation of transcriptio
GO:0006633-fatty acid biosynthetic process
GO:0035556-intracellular signal transduction
GO:0018105-peptidyl-serine phosphorylation
GO:0009733-response to auxin
GO:0009414-response to water deprivation
GO:0006357-regulation of transcription by RN
GO:0051301-cell division
GO:0016567-protein ubiquitination
GO:0043161-proteasome-mediated ubiquitin-d
GO:0006096-glycolytic process
GO:0002181-cytoplasmic translation
GO:0006511-ubiquitin-dependent protein catat
GO:0000398-mRNA splicing, via spliceosome
GO:0009409-response to cold
GO:0009408-response to heat
GO:0009735-response to cytokinin
GO:0018298-protein-chromophore linkage
GO:0009768-photosynthesis, light harvesting i
GO:0009416-response to light stimulus
GO:0006457-protein folding
GO:0006412-translation
S._lyc-8_vs_0
S._arc-8_vs_0
S._arc-24_vs_8
S._lyc-24_vs_8

(b)

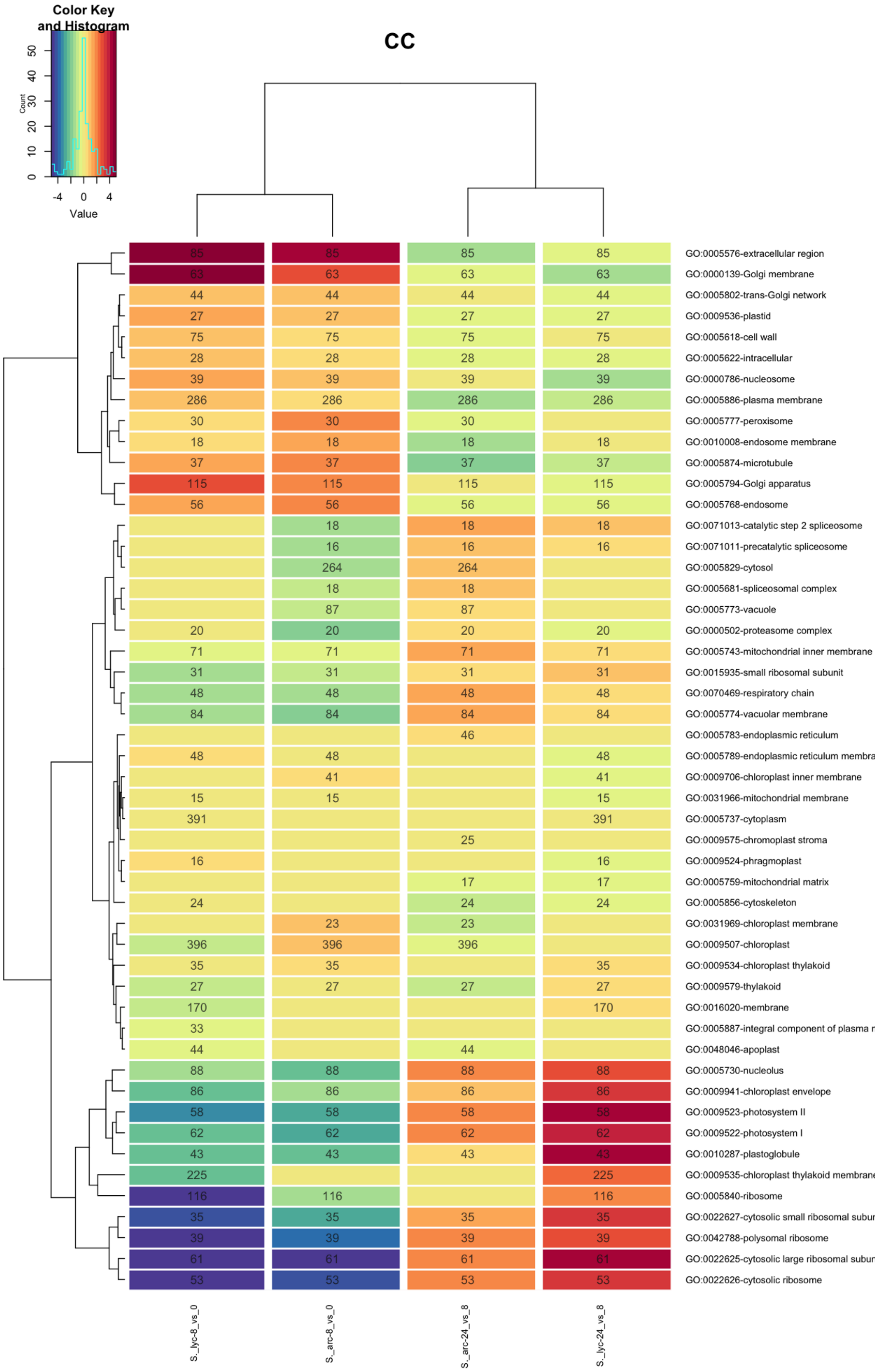
Color Key
and Histogram
Count
Value
CC
GO:0005576-extracellular region
GO:0000139-Golgi membrane
GO:0005802-trans-Golgi network
GO:0009536-plastid
GO:0005618-cell wall
GO:0005622-intracellular
GO:0000786-nucleosome
GO:0005886-plasma membrane
GO:0005777-peroxisome
GO:0010008-endosome membrane
GO:0005874-microtubule
GO:0005794-Golgi apparatus
GO:0005768-endosome
GO:0071013-catalytic step 2 spliceosome
GO:0071011-precatalytic spliceosome
GO:0005829-cytosol
GO:0005681-spliceosomal complex
GO:0005773-vacuole
GO:0000502-proteasome complex
GO:0005743-mitochondrial inner membrane
GO:0015935-small ribosomal subunit
GO:0070469-respiratory chain
GO:0005774-vacuolar membrane
GO:0005783-endoplasmic reticulum
GO:0005789-endoplasmic reticulum membra
GO:0009706-chloroplast inner membrane
GO:0031966-mitochondrial membrane
GO:0005737-cytoplasm
GO:0009575-chromoplast stroma
GO:0009524-phragmoplast
GO:0005759-mitochondrial matrix
GO:0005856-cytoskeleton
GO:0031969-chloroplast membrane
GO:0009507-chloroplast
GO:0009534-chloroplast thylakoid
GO:0009579-thylakoid
GO:0016020-membrane
GO:0005887-integral component of plasma r
GO:0048046-apoplast
GO:0005730-nucleolus
GO:0009941-chloroplast envelope
GO:0009523-photosystem II
GO:0009522-photosystem I
GO:0010287-plastoglobule
GO:0009535-chloroplast thylakoid membrane
GO:0005840-ribosome
GO:0022627-cytosolic small ribosomal subur
GO:0042788-polysomal ribosome
GO:0022625-cytosolic large ribosomal subur
GO:0022626-cytosolic ribosome
S._lyc-8_vs_0
S._arc-8_vs_0
S._arc-24_vs_8
S._lyc-24_vs_8

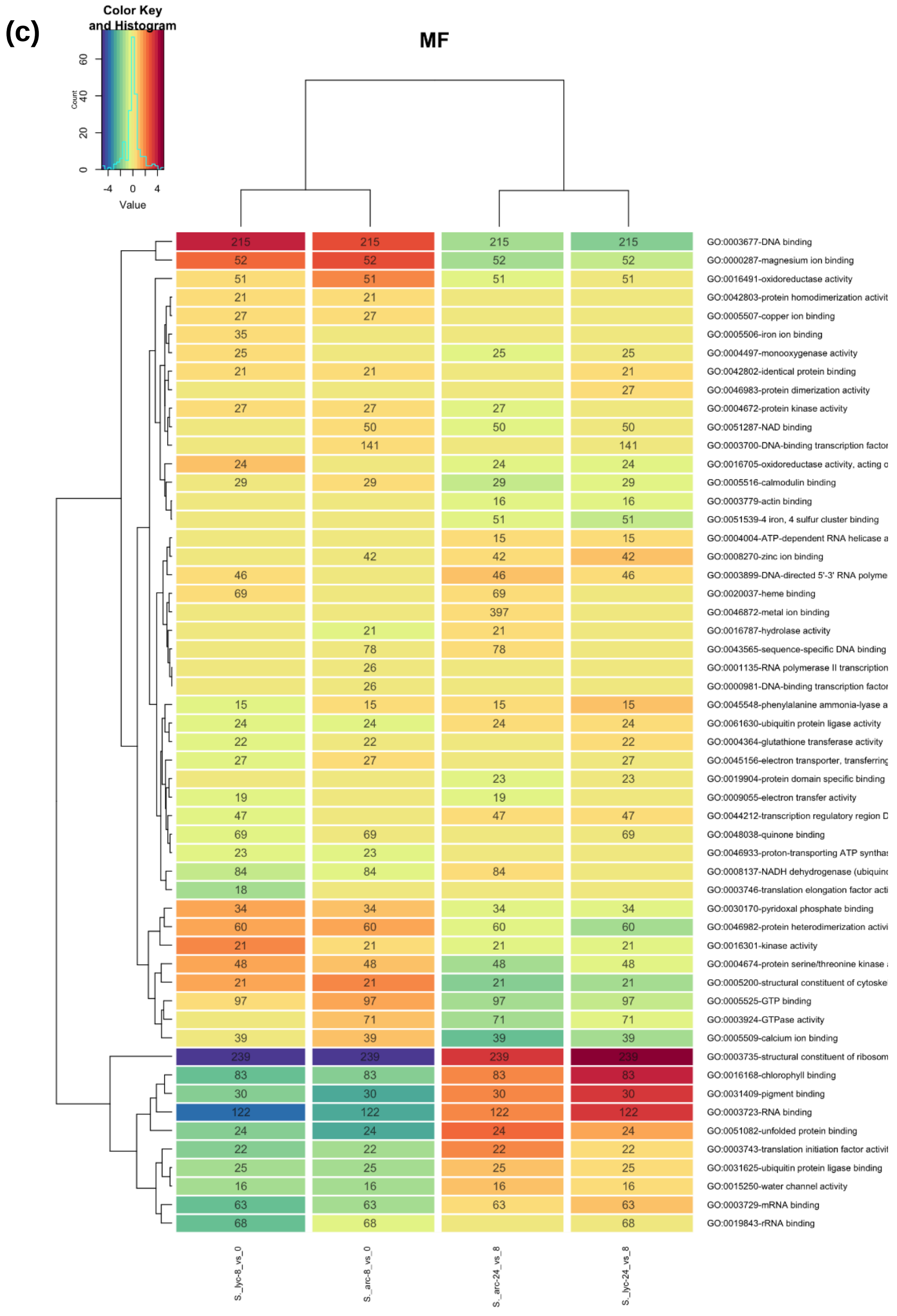

Figure S2. GO terms enrichment resulting from MR analysis. (a) Enriched functional groups in biological process ontology (BP). (b) Enriched functional groups in cellular component ontology (CC). (c) Enriched functional groups in molecular function ontology (MF). GO term enrichment was performed using a logistic regression comparing scores of the genes/transcripts. Scores were calculated from LogFC and the –log10 of FDR values.

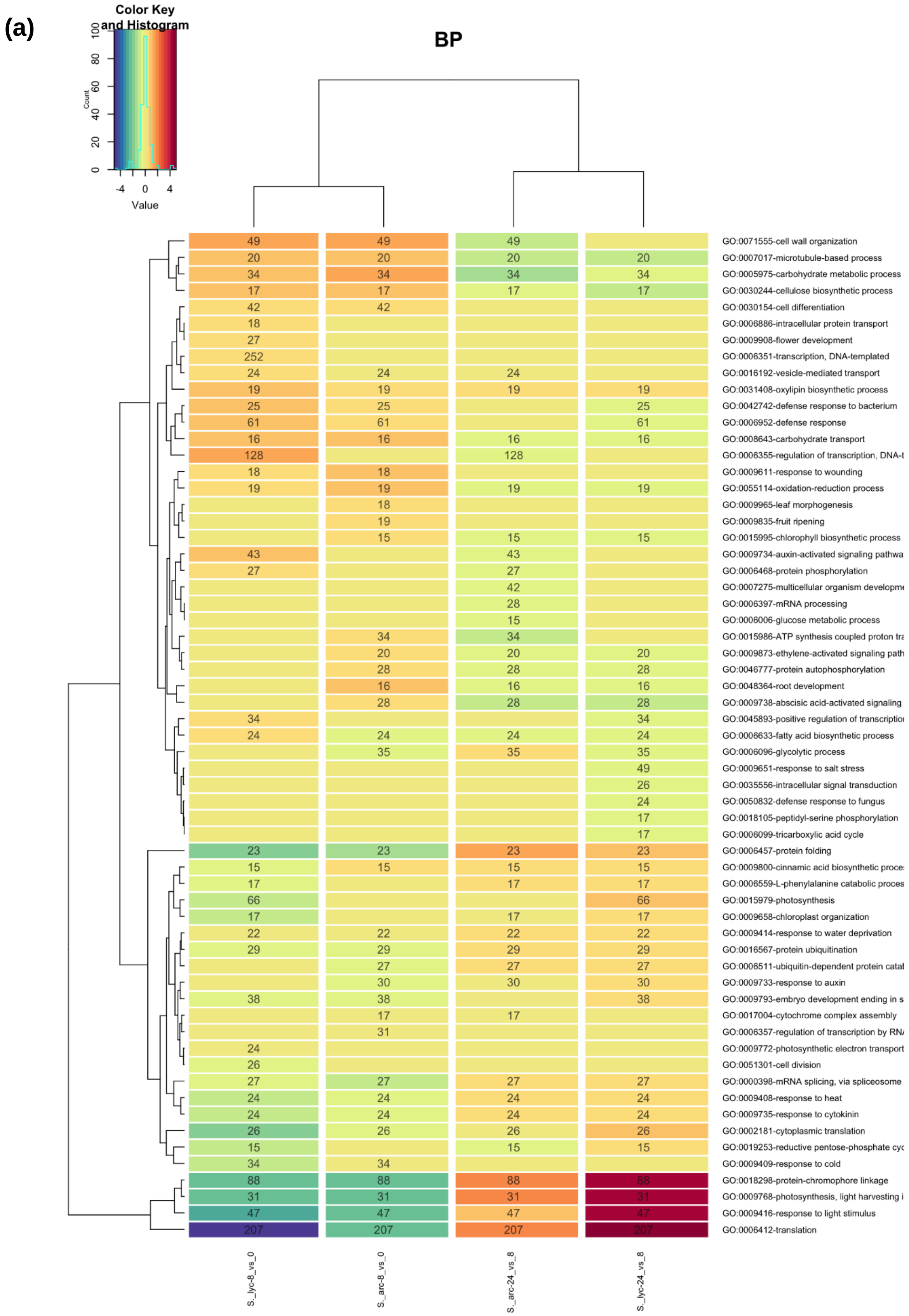
(a)
Color Key and Histogram
BP
Count
Value
S_lyc-8_vs_0
S_arc-8_vs_0
S_arc-24_vs_8
S_lyc-24_vs_8
GO:0071555-cell wall organization
GO:0007017-microtubule-based process
GO:0005975-carbohydrate metabolic process
GO:0030244-cellulose biosynthetic process
GO:0030154-cell differentiation
GO:0006886-intracellular protein transport
GO:0009908-flower development
GO:0006351-transcription, DNA-templated
GO:0016192-vesicle-mediated transport
GO:0031408-oxylipin biosynthetic process
GO:0042742-defense response to bacterium
GO:0006952-defense response
GO:0008643-carbohydrate transport
GO:0006355-regulation of transcription, DNA-t
GO:0009611-response to wounding
GO:0055114-oxidation-reduction process
GO:0009965-leaf morphogenesis
GO:0009835-fruit ripening
GO:0015995-chlorophyll biosynthetic process
GO:0009734-auxin-activated signaling pathwa
GO:0006468-protein phosphorylation
GO:0007275-multicellular organism developme
GO:0006397-mRNA processing
GO:0006006-glucose metabolic process
GO:0015986-ATP synthesis coupled proton tra
GO:0009873-ethylene-activated signaling path
GO:0046777-protein autophosphorylation
GO:0048364-root development
GO:0009738-abscisic acid-activated signaling
GO:0045893-positive regulation of transcriptio
GO:0006633-fatty acid biosynthetic process
GO:0006096-glycolytic process
GO:0009651-response to salt stress
GO:0035556-intracellular signal transduction
GO:0050832-defense response to fungus
GO:0018105-peptidyl-serine phosphorylation
GO:0006099-tricarboxylic acid cycle
GO:0006457-protein folding
GO:0009800-cinnamic acid biosynthetic proce
GO:0006559-L-phenylalanine catabolic proces
GO:0015979-photosynthesis
GO:0009658-chloroplast organization
GO:0009414-response to water deprivation
GO:0016567-protein ubiquitination
GO:0006511-ubiquitin-dependent protein catat
GO:0009733-response to auxin
GO:0009793-embryo development ending in s
GO:0017004-cytochrome complex assembly
GO:0006357-regulation of transcription by RN
GO:0009772-photosynthetic electron transport
GO:0051301-cell division
GO:0000398-mRNA splicing, via spliceosome
GO:0009408-response to heat
GO:0009735-response to cytokinin
GO:0002181-cytoplasmic translation
GO:0019253-reductive pentose-phosphate cyc
GO:0009409-response to cold
GO:0018298-protein-chromophore linkage
GO:0009768-photosynthesis, light harvesting i
GO:0009416-response to light stimulus
GO:0006412-translation

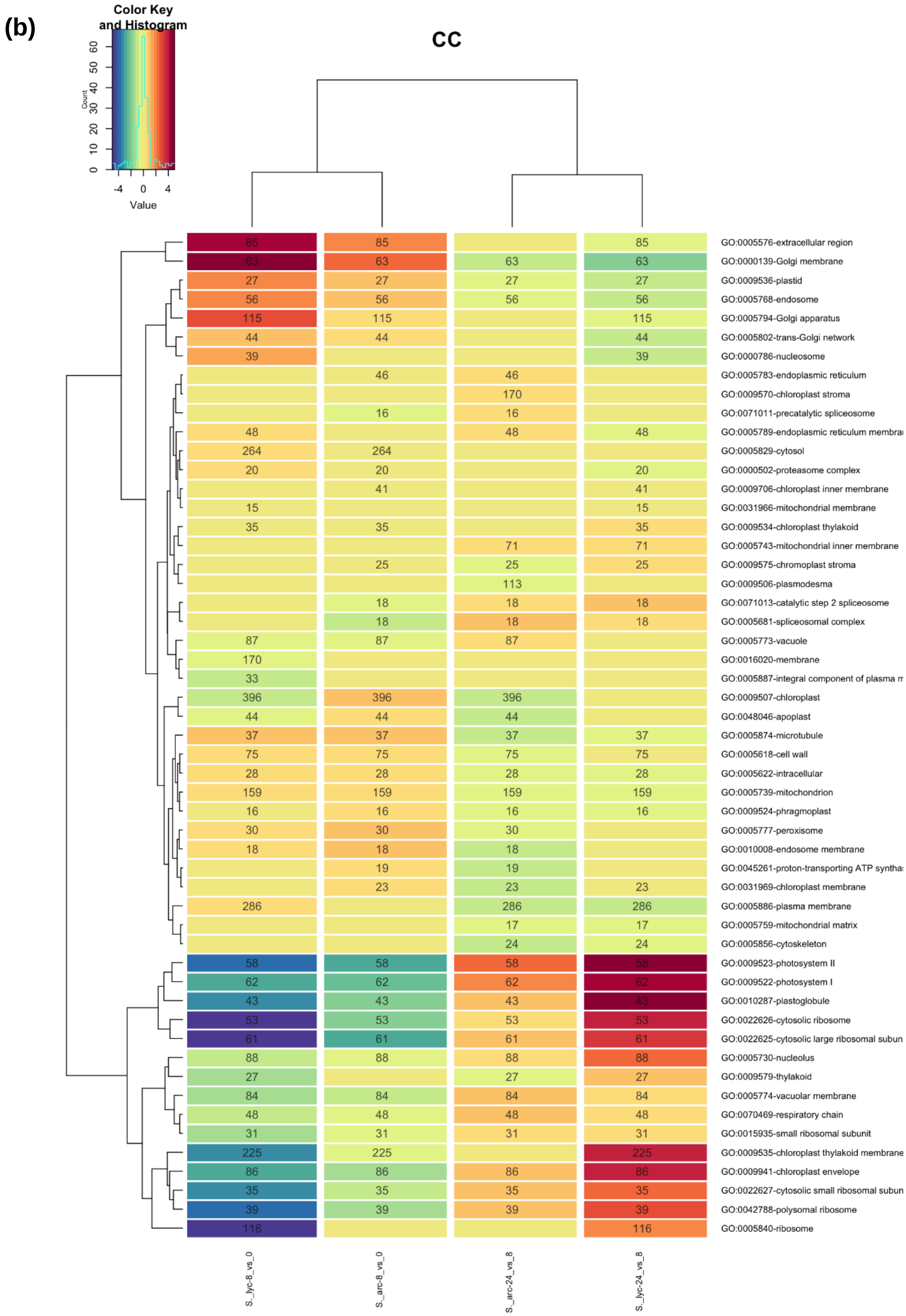
(b)
Color Key and Histogram
Count
Value
CC
GO:0005576-extracellular region
GO:0000139-Golgi membrane
GO:0009536-plastid
GO:0005768-endosome
GO:0005794-Golgi apparatus
GO:0005802-trans-Golgi network
GO:0000786-nucleosome
GO:0005783-endoplasmic reticulum
GO:0009570-chloroplast stroma
GO:0071011-precatalytic spliceosome
GO:0005789-endoplasmic reticulum membrai
GO:0005829-cytosol
GO:0000502-proteasome complex
GO:0009706-chloroplast inner membrane
GO:0031966-mitochondrial membrane
GO:0009534-chloroplast thylakoid
GO:0005743-mitochondrial inner membrane
GO:0009575-chromoplast stroma
GO:0009506-plasmodesma
GO:0071013-catalytic step 2 spliceosome
GO:0005681-spliceosomal complex
GO:0005773-vacuole
GO:0016020-membrane
GO:0005887-integral component of plasma m
GO:0009507-chloroplast
GO:0048046-apoplast
GO:0005874-microtubule
GO:0005618-cell wall
GO:0005622-intracellular
GO:0005739-mitochondrion
GO:0009524-phragmoplast
GO:0005777-peroxisome
GO:0010008-endosome membrane
GO:0045261-proton-transporting ATP syntha:
GO:0031969-chloroplast membrane
GO:0005886-plasma membrane
GO:0005759-mitochondrial matrix
GO:0005856-cytoskeleton
GO:0009523-photosystem II
GO:0009522-photosystem I
GO:0010287-plastoglobule
GO:0022626-cytosolic ribosome
GO:0022625-cytosolic large ribosomal subun
GO:0005730-nucleolus
GO:0009579-thylakoid
GO:0005774-vacuolar membrane
GO:0070469-respiratory chain
GO:0015935-small ribosomal subunit
GO:0009535-chloroplast thylakoid membrane
GO:0009941-chloroplast envelope
GO:0022627-cytosolic small ribosomal subun
GO:0042788-polysomal ribosome
GO:0005840-ribosome
S._lyc-8_vs_0
S._arc-8_vs_0
S._arc-24_vs_8
S._lyc-24_vs_8

(c)

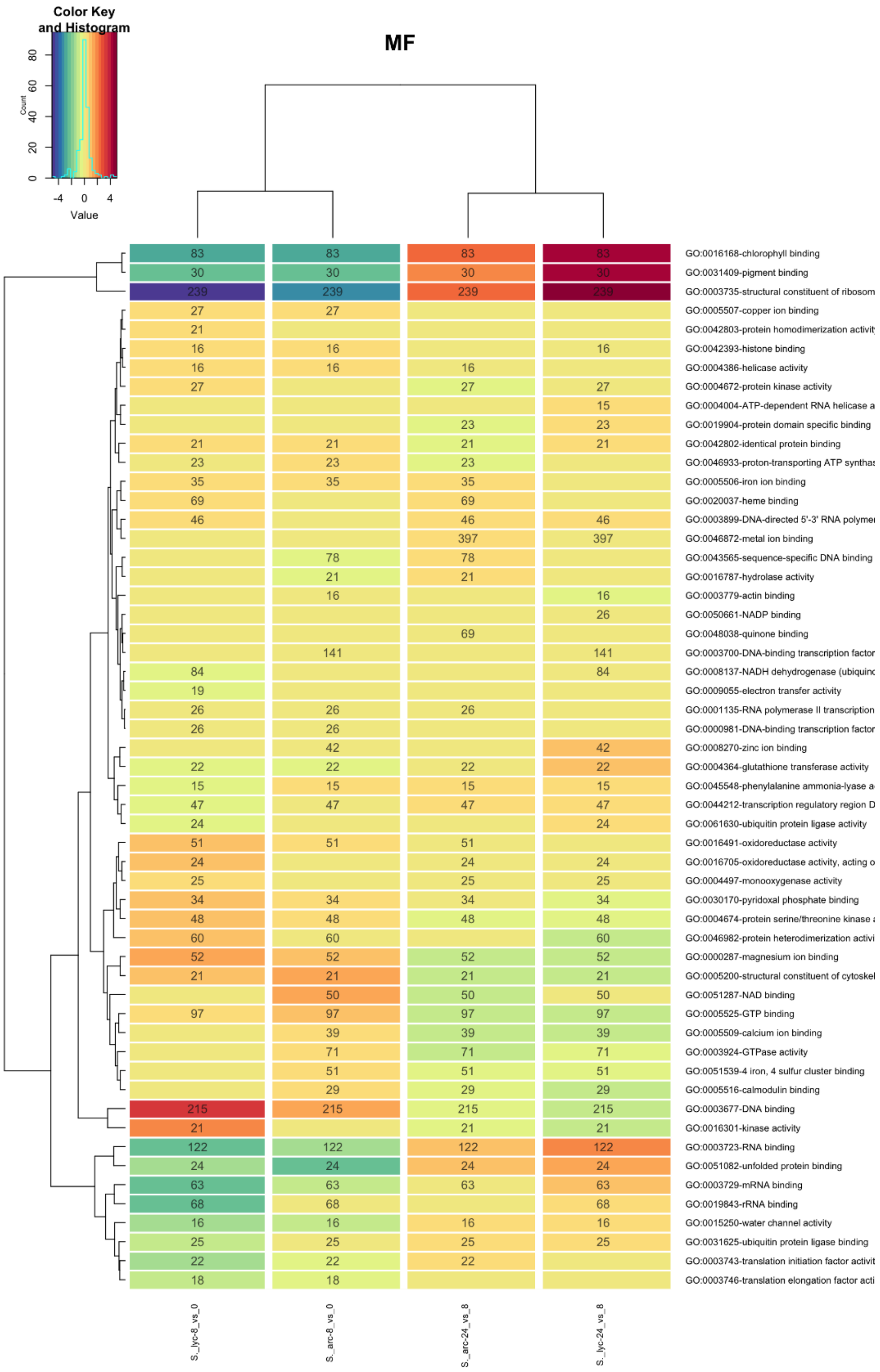

Figure S3. GO terms enrichment resulting from STA analysis. (a) Enriched functional groups in biological process ontology (BP). (b) Enriched functional groups in cellular component ontology (CC). (c) Enriched functional groups in molecular function ontology (MF). GO term enrichment was performed using a logistic regression comparing scores of the genes/transcripts. Scores were calculated from LogFC and the –log10 of FDR values.

(a)

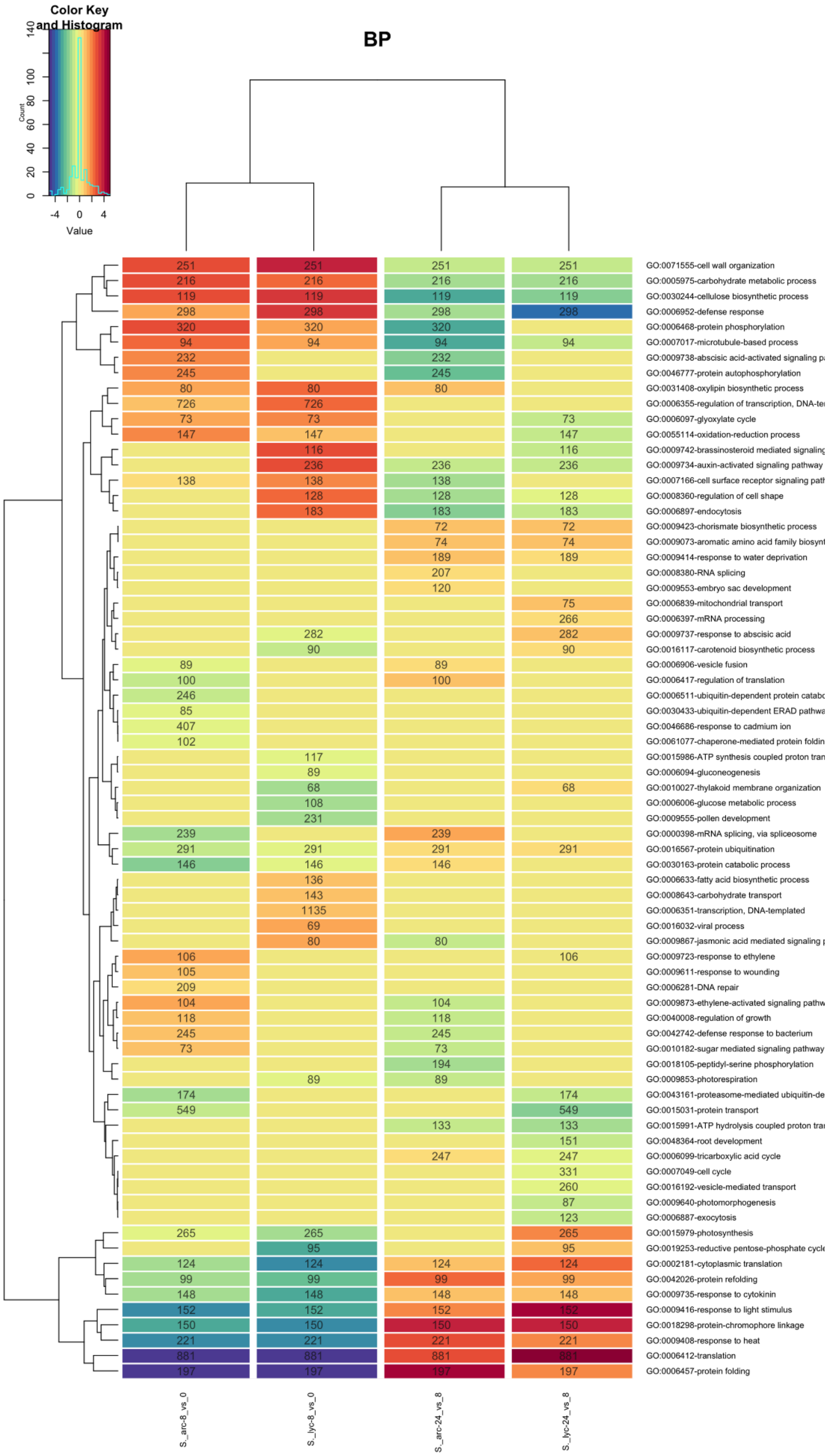

Color Key and Histogram
Count
Value
BP
S._arc-8_vs_0
S._lyc-8_vs_0
S._arc-24_vs_8
S._lyc-24_vs_8
GO:0071555-cell wall organization
GO:0005975-carbohydrate metabolic process
GO:0030244-cellulose biosynthetic process
GO:0006952-defense response
GO:0006468-protein phosphorylation
GO:0007017-microtubule-based process
GO:0046777-protein autophosphorylation
GO:0031408-oxylipin biosynthetic process
GO:0006097-glyoxylate cycle
GO:0055114-oxidation-reduction process
GO:0009734-auxin-activated signaling pathway
GO:0008360-regulation of cell shape
GO:0006897-endocytosis
GO:0009423-chorismate biosynthetic process
GO:0009414-response to water deprivation
GO:0008380-RNA splicing
GO:0009553-embryo sac development
GO:0006839-mitochondrial transport
GO:0006397-mRNA processing
GO:0009737-response to abscisic acid
GO:0016117-carotenoid biosynthetic process
GO:0006906-vesicle fusion
GO:0006417-regulation of translation
GO:0046686-response to cadmium ion
GO:0006094-gluconeogenesis
GO:0010027-thylakoid membrane organization
GO:0006006-glucose metabolic process
GO:0009555-pollen development
GO:0000398-mRNA splicing, via spliceosome
GO:0016567-protein ubiquitination
GO:0030163-protein catabolic process
GO:0006633-fatty acid biosynthetic process
GO:0008643-carbohydrate transport
GO:0006351-transcription, DNA-templated
GO:0016032-viral process
GO:0009723-response to ethylene
GO:0009611-response to wounding
GO:0006281-DNA repair
GO:0040008-regulation of growth
GO:0042742-defense response to bacterium
GO:0010182-sugar mediated signaling pathway
GO:0018105-peptidyl-serine phosphorylation
GO:0009853-photorespiration
GO:0015031-protein transport
GO:0048364-root development
GO:0006099-tricarboxylic acid cycle
GO:0007049-cell cycle
GO:0016192-vesicle-mediated transport
GO:0009640-photomorphogenesis
GO:0006887-exocytosis
GO:0015979-photosynthesis
GO:0002181-cytoplasmic translation
GO:0042026-protein refolding
GO:0009735-response to cytokinin
GO:0009416-response to light stimulus
GO:0018298-protein-chromophore linkage
GO:0009408-response to heat
GO:0006412-translation
GO:0006457-protein folding

(b)

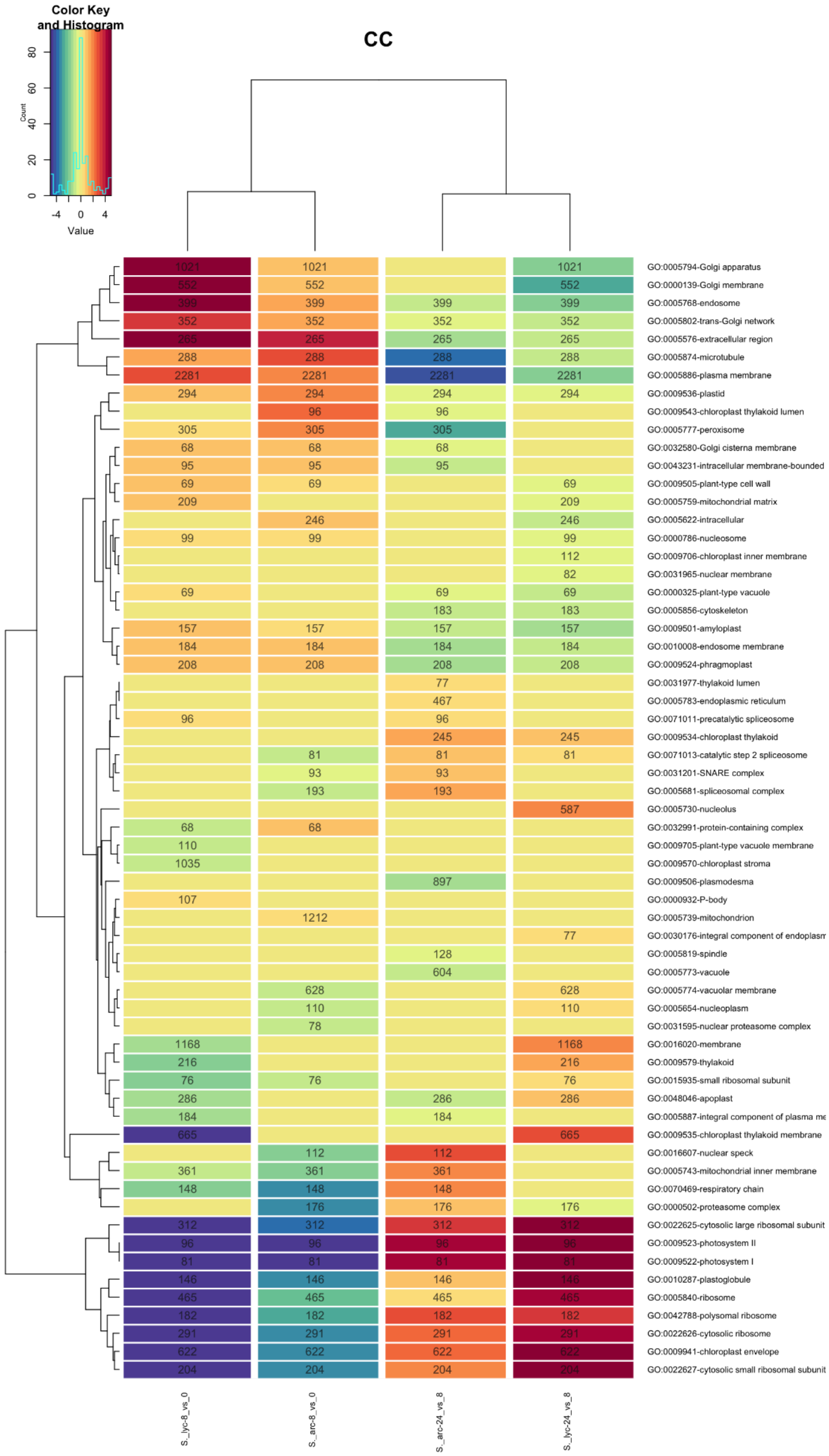

CC
Color Key and Histogram
Count
Value
GO:0005794-Golgi apparatus
GO:0000139-Golgi membrane
GO:0005768-endosome
GO:0005802-trans-Golgi network
GO:0005576-extracellular region
GO:0005874-microtubule
GO:0005886-plasma membrane
GO:0009536-plastid
GO:0009543-chloroplast thylakoid lumen
GO:0005777-peroxisome
GO:0032580-Golgi cisterna membrane
GO:0043231-intracellular membrane-bounded
GO:0009505-plant-type cell wall
GO:0005759-mitochondrial matrix
GO:0005622-intracellular
GO:0000786-nucleosome
GO:0009706-chloroplast inner membrane
GO:0031965-nuclear membrane
GO:0000325-plant-type vacuole
GO:0005856-cytoskeleton
GO:0009501-amyloplast
GO:0010008-endosome membrane
GO:0009524-phragmoplast
GO:0031977-thylakoid lumen
GO:0005783-endoplasmic reticulum
GO:0071011-precatalytic spliceosome
GO:0009534-chloroplast thylakoid
GO:0071013-catalytic step 2 spliceosome
GO:0031201-SNARE complex
GO:0005681-spliceosomal complex
GO:0005730-nucleolus
GO:0032991-protein-containing complex
GO:0009705-plant-type vacuole membrane
GO:0009570-chloroplast stroma
GO:0009506-plasmodesma
GO:0000932-P-body
GO:0005739-mitochondrion
GO:0030176-integral component of endoplasm
GO:0005819-spindle
GO:0005773-vacuole
GO:0005774-vacuolar membrane
GO:0005654-nucleoplasm
GO:0031595-nuclear proteasome complex
GO:0016020-membrane
GO:0009579-thylakoid
GO:0015935-small ribosomal subunit
GO:0048046-apoplast
GO:0005887-integral component of plasma me
GO:0009535-chloroplast thylakoid membrane
GO:0016607-nuclear speck
GO:0005743-mitochondrial inner membrane
GO:0070469-respiratory chain
GO:0000502-proteasome complex
GO:0022625-cytosolic large ribosomal subunit
GO:0009523-photosystem II
GO:0009522-photosystem I
GO:0010287-plastoglobule
GO:0005840-ribosome
GO:0042788-polysomal ribosome
GO:0022626-cytosolic ribosome
GO:0009941-chloroplast envelope
GO:0022627-cytosolic small ribosomal subunit
S._lyc-8_vs_0
S._arc-8_vs_0
S._arc-24_vs_8
S._lyc-24_vs_8

(c)

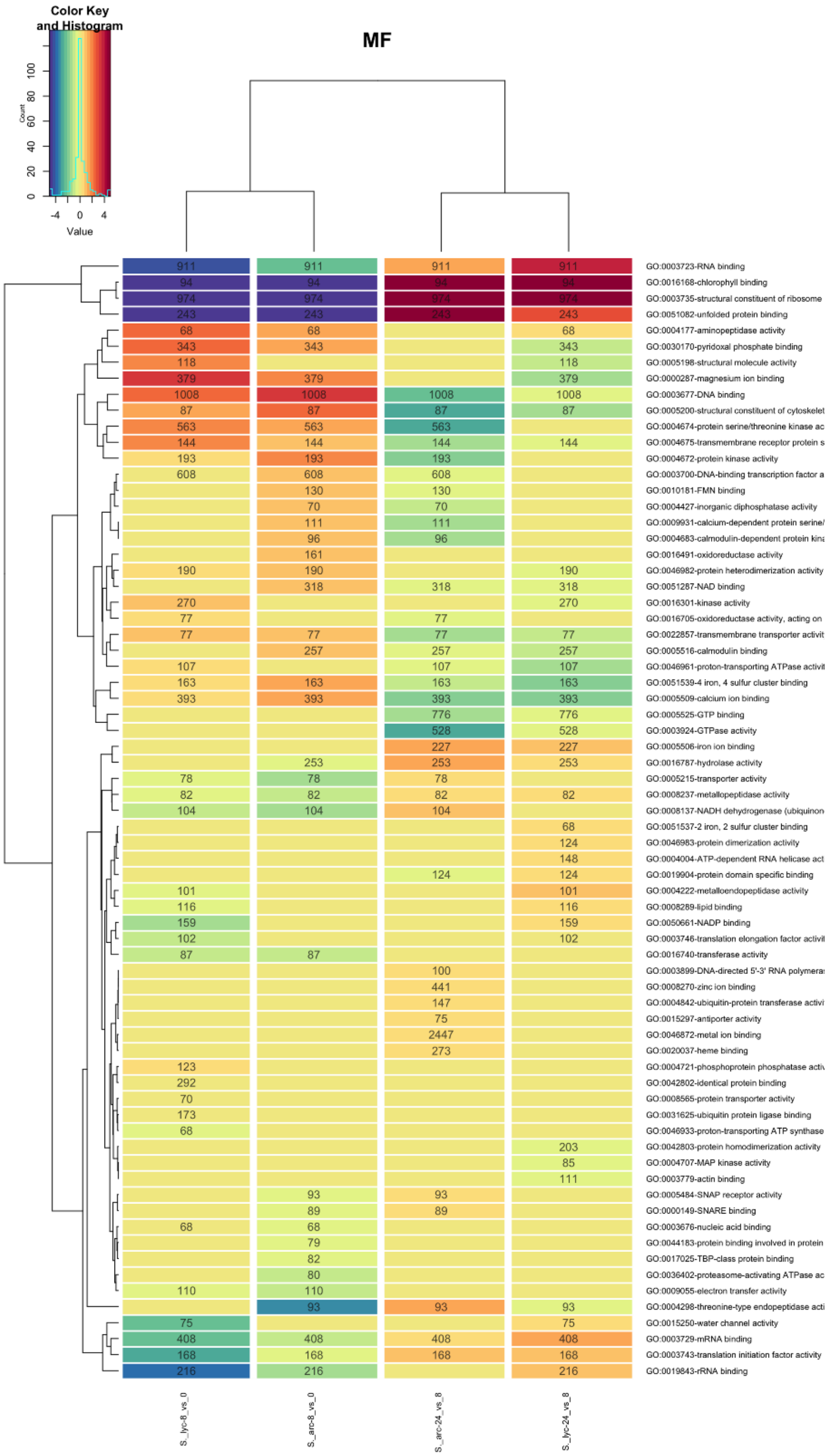

Figure S4. GO terms enrichment resulting from DA analysis. (a) Enriched functional groups in biological process ontology (BP). (b) Enriched functional groups in cellular component ontology (CC). (c) Enriched functional groups in molecular function ontology (MF). GO term enrichment was performed using a logistic regression comparing scores of the genes/transcripts. Scores were calculated from LogFC and the –log10 of FDR values.

Table S1. Average computing time consumed per strategy.

Approach	Assembly	Annotation	Mapping & quantification (per library)	Total time
**Mapping to the reference genome SL3.0**	---	---	~ 3 h	~ 3 h
**Semi *de novo* assembly**	~ 7 h	~ 15 h	~ 3 h	~ 25 h
***De novo* assembly**	~ 240 h	~ 38 h	~ 1 h	~ 279 h

Note: This analysis was carried out in a cluster (Thubat Kaal 2.0), with 18 FAT nodes and 400GB in RAM with 32 processors.

Table S2. Main features of generated transcriptomes.

Approach	Total transcripts	%GC content	N50	N90	Total bases	Predicted transcripts	Predicted transcripts per gene
**Mapping to the reference genome SL3.0**	35768	39.12	2226	801	54440110	0	1
**Semi *de novo* assembly**	77515	39.47	1157	581	166329048	123697	2.057214733
***De novo* assembly**	49340	38.62	2031	829	286104977	176548	3.578192136

Table S9. List of primers employed for qRT-PCR validation.

Name	Primer Sequence (5´- 3')	Length (bp)	Amplicon size (bp)
**PPO-E**	F: TTGGTAAGGAAGTTGACACACC	22	187
	R: CAGATGTGAACCGGAGTATGAG	22	
**LRR-At1g17230**	F: GTTGCTGGTTCTGTCGGTTAT	21	183
	R: CCACTTGCTGTGCTTCTCAG	20	
**Ankyrin**	F: GGTAATGGACACAACATTCTGC	22	162
	R: TAGATCATTCACGTCCCTACGA	22	
**PHLOEM PL2**	F: GGAGAAATAGGAACTGGGTTGT	22	150
	R: CCACCATCTCAGCTAAAGAAAC	22	
**MACPF**	F: CTTACTCATCTCGGTCATGTCA	22	183
	R: CTTCTGTATTCAAGGCTACCAG	22	
**ACT-TOM5**	F: CCTCACCGAGAGAGGTTACATGT	23	61
	R: CATGTCGCGGACAATTTCC	19	

F: Forward primer; R: Reverse primer.
